# Correlative analysis from a phase I clinical trial of intrapleural administration of oncolytic vaccinia virus (Olvi-vec) in patients with malignant pleural mesothelioma

**DOI:** 10.3389/fimmu.2023.1112960

**Published:** 2023-02-16

**Authors:** Navin K. Chintala, Jennie K. Choe, Erin McGee, Rebecca Bellis, Jasmeen K. Saini, Srijita Banerjee, Andre L. Moreira, Marjorie G. Zauderer, Prasad S. Adusumilli, Valerie W. Rusch

**Affiliations:** ^1^ Thoracic Service, Department of Surgery, Memorial Sloan Kettering Cancer Center, New York, NY, United States; ^2^ Department of Pathology, New York University (NYU) Grossman School of Medicine, New York, NY, United States; ^3^ Thoracic Oncology Service, Department of Medicine, Memorial Sloan Kettering Cancer Center, New York, NY, United States; ^4^ Center for Cell Engineering, Memorial Sloan Kettering Cancer Center, New York, NY, United States

**Keywords:** pleural cancers, oncolytic viral therapy, tumor microenvironment, regional therapy, malignant pleural effusion (MPE)

## Abstract

**Background:**

The attenuated, genetically engineered vaccinia virus has been shown to be a promising oncolytic virus for the treatment of patients with solid tumors, through both direct cytotoxic and immune-activating effects. Whereas systemically administered oncolytic viruses can be neutralized by pre-existing antibodies, locoregionally administered viruses can infect tumor cells and generate immune responses. We conducted a phase I clinical trial to investigate the safety, feasibility and immune activating effects of intrapleural administration of oncolytic vaccinia virus (NCT01766739).

**Methods:**

Eighteen patients with malignant pleural effusion due to either malignant pleural mesothelioma or metastatic disease (non-small cell lung cancer or breast cancer) underwent intrapleural administration of the oncolytic vaccinia virus using a dose-escalating method, following drainage of malignant pleural effusion. The primary objective of this trial was to determine a recommended dose of attenuated vaccinia virus. The secondary objectives were to assess feasibility, safety and tolerability; evaluate viral presence in the tumor and serum as well as viral shedding in pleural fluid, sputum, and urine; and evaluate anti-vaccinia virus immune response. Correlative analyses were performed on body fluids, peripheral blood, and tumor specimens obtained from pre- and post-treatment timepoints.

**Results:**

Treatment with attenuated vaccinia virus at the dose of 1.00E+07 plaque-forming units (PFU) to 6.00E+09 PFU was feasible and safe, with no treatment-associated mortalities or dose-limiting toxicities. Vaccinia virus was detectable in tumor cells 2-5 days post-treatment, and treatment was associated with a decrease in tumor cell density and an increase in immune cell density as assessed by a pathologist blinded to the clinical observations. An increase in both effector (CD8+, NK, cytotoxic cells) and suppressor (Tregs) immune cell populations was observed following treatment. Dendritic cell and neutrophil populations were also increased, and immune effector and immune checkpoint proteins (granzyme B, perforin, PD-1, PD-L1, and PD-L2) and cytokines (IFN-γ, TNF-α, TGFβ1 and RANTES) were upregulated.

**Conclusion:**

The intrapleural administration of oncolytic vaccinia viral therapy is safe and feasible and generates regional immune response without overt systemic symptoms.

**Clinical trial registration:**

https://clinicaltrials.gov/ct2/show/NCT01766739, identifier NCT01766739.

## Introduction

With an estimated annual incidence of at least 150,000 patients in the United States, malignant pleural effusions (MPEs) occur in 15% of all patients with cancer during the course of their disease (1). In addition to causing symptoms that limit quality of life, such as shortness of breath that requires interventions (2), the presence of MPE represents advanced cancer and contributes to poor prognosis (3). Palliative interventions have been the mainstay for symptomatic relief and prevention of MPE recurrence that can interrupt cancer therapy in patients with MPE (2, 4). There has been limited success following systemic immune checkpoint inhibitor agent therapy, chemotherapy or a combination of chemo immunotherapies in patients with MPE (5, 6). Intrapleural biological therapies, such as oncolytic viral therapy and chimeric antigen receptor (CAR) T-cell therapy, have been investigated to promote effector immune responses in patients with MPEs (7–10). However, local immune suppressive mechanisms in MPEs that inhibit the efficacy of effector immune responses have been well described (11, 12). Specifically, macrophages and TGFβ in MPEs have been shown to play a pivotal role in hampering the antitumor immune responses (11, 13). Correlative analysis of pre- and post-treatment MPEs and pleural tumor biopsies to characterize the effector and suppressor immune responses following intrapleural therapies can shed light on changes in the immune microenvironment and aid in developing regimens to further enhance functional efficacy.

Malignant pleural mesothelioma (MPM), metastatic non-small cell lung cancer, and breast cancers are common causes of MPEs. Malignant pleural mesothelioma is a rare cancer with diffuse involvement of the pleural cavity. Immune checkpoint inhibitors have shown promising results in patients with MPM; however, the increase in survival is limited to mostly biphasic and sarcomatoid forms of MPM (14, 15). Epithelioid MPM, the most common form of MPM, is known to have the lowest tumor mutational burden and PD-L1 expression among solid tumors (16), with equivalent survival compared to chemotherapy (15). Regional oncolytic viral therapies that can generate effector immune responses in patients with MPM may provide an opportunity for subsequent immune checkpoint inhibitor agent therapy (17).

Oncolytic viruses, which selectively infect and exert cytopathic effects on tumor cells, are a potential therapeutic option for MPM. As a member of the poxvirus family of the genus orthopoxvirus, the vaccinia virus is one such oncolytic virus that possesses multiple favorable features for use as a therapeutic agent. It exhibits rapid cell-to-cell spread, is cytolytic across a broad range of tumor cell types, has a large insertion capacity for exogenous genes, and is genetically stable with low potential for mutagenesis (18). It is amenable to large-scale manufacture, storage and production, and is safe to administer intravenously (19).

In *in vitro* studies, the vaccinia virus has shown to be efficient in killing multiple cancer cell lines, including breast, lung, thyroid, prostate, pancreas, squamous cell carcinoma, and MPM (20). In *in vivo* studies, the vaccinia virus has caused tumor elimination in mouse models of breast cancer and MPM, as well as tumor growth inhibition in mouse models of lung adenocarcinoma, anaplastic thyroid cancer (21), prostate cancer (22), ovarian cancer, pancreatic cancer, and melanoma (23). Isolated case reports have documented complete remission in a patient with multiple myeloma (24) and a patient with chronic lymphocytic leukemia (25, 26) following vaccinia virus administration. Vaccinia virus has also been used in phase I clinical trials to treat patients with bladder cancer (27), metastatic melanoma (28), and advanced hepatocellular carcinoma (29, 30).

We conducted a single-center phase I clinical trial (NCT01766739) to study the intrapleural administration of attenuated vaccinia virus (GL-ONC1, Genelux Corporation) in patients with MPEs due to MPM or metastatic disease (non-small cell lung cancer or breast cancer). In 2019, the United States Adopted Names Council (USAN) granted Genelux adoption of the name *Olvimulogene nanivacirepvec* (referred to as Olvi-vec) in place of the name GL-ONC1; henceforth referred to as Olvi-vec throughout the manuscript.

## Materials and methods

### Trial design and patients

An open-label, dose-escalating, non-randomized, single-center phase I study was conducted to study the intrapleural administration of attenuated vaccinia virus (Olvi-vec) as a bolus. Olvi-vec was administered either as a single dose or as three consecutive daily doses to patients with a histologically or cytologically documented diagnosis of MPE, as detailed in the study protocol (**Supplementary Material**).

### Study oversight

The study protocol and amendments were approved by the Memorial Sloan Kettering Cancer Center Institutional Review Board (IRB# 12-169, NCT01766739). All patients provided written informed consent to participate in the study, and all response and toxicity outcomes were documented. Patients were enrolled in groups of three and individually assessed for safety and dose-limiting toxicity. Inclusion and exclusion criteria are listed in the protocol (**Supplementary Material**). Patients were treated following the diagnosis of histologically or cytologically documented MPEs (due to primary non-small-cell lung carcinoma, MPM, and other histologies) and had free pleural space (partial or total) that permitted intrapleural drug instillation.

### Olvi-vec manufacturing

A genetically engineered vaccinia virus, designated as GLV-1h68, was used in preclinical investigation. GLV-1h68 was derived from the LIVP strain by inserting *RUC-GFP* (a fusion gene of *Renilla* luciferase and green fluorescent protein), *LacZ* (beta-galactosidase), and *gusA* (beta-glucuronidase) expression cassettes into *F14.5L* (located between *F14L* and *F15L*), thymidine kinase (TK), and hemagglutinin loci, respectively. Disruption of these non-essential genes and expression of the foreign gene expression cassettes not only attenuated the virus but also enhanced its tumor-specific targeting. The GMP-derived material of this same virus is called Olvi-vec. Olvi-vec has been used primarily for all safety pharmacology and toxicological experiments, as well as for *in vitro* potency comparisons (in cell cultures) and *in vivo* potency comparisons (in tumorous animals). Details of the virus manufacturing process and analyses are described in the study protocol (**Supplementary Material**).

### Intrapleural treatment

Eligible patients were admitted into the hospital for treatment on protocol. The pleural effusion was drained *via* insertion of a chest tube or pleural catheter (PleurX™ Catheter, Becton, Dickinson and Company, Franklin Lakes, NJ). A chest CT scan was performed to document drainage of the effusion and to assess the extent of pleural disease. Within 72 hours of the CT scan, the virus was instilled as a bolus into the pleural space *via* the chest tube or pleural catheter. Up to 150 ml of additional saline was used to flush the chest tube or pleural catheter to ensure that all the treatment drug was instilled into the pleural space. The chest tube or pleural catheter was left clamped for 4 hours (+/- 1 hour), after which it was reopened and placed to drainage in order to drain the pleural space. As dictated by the patient’s clinical status, the chest tube was either left inserted or removed until the surgical procedure (video-assisted thoracoscopic surgery, VATS) was performed 2-7 days after treatment to collect MPE and obtain pleural biopsy.

### Study objectives and assessment

The primary objective of this study was to determine a recommended dose of Olvi-vec. The secondary objectives included the assessment of feasibility, safety and tolerability, evaluation of viral presence in the tumor, pleural fluid, serum, sputum, and urine, and evaluation of anti-vaccinia virus immune response. All patients were included in the reporting of adverse events (AEs). The safety of Olvi-vec was assessed by the evaluation of the type, frequency, and severity of AEs, changes in clinical laboratory tests (hematological and chemistry), immunogenicity, and physical examination. All AEs and laboratory toxicities were graded using the Common Terminology Criteria for Adverse Events (National Cancer Institute, version 4.0). Laboratory testing was performed at baseline (i.e., within 14 days before treatment), daily during the first 3 days after treatment, and at termination of study (day 60 ± 5).

### Hematoxylin and eosin staining

Hematoxylin and eosin staining was performed on FFPE blocks of tumor biopsies collected before (pre-treatment) and 2-5 days after (post-treatment) Olvi-vec therapy. Semi-quantitative scoring of tumor cell density and immune cell density (0: very low density; 1: low density; 2: moderate density; 3: high density) was performed by a primary and secondary pathologist who were blinded to sample identity.

### Multiplex immunofluorescence staining

Multiplex immunofluorescence (mIF) staining was performed on tumor biopsies collected before (pre-treatment) and 2-5 days after (post-treatment) Olvi-vec therapy. Formalin-fixed and paraffin-embedded (FFPE) blocks were cut into sections of 5 µm thickness. Sections from each biopsy were stained with antibodies (**Supplementary Table 1**) using the Opal™ 7-Color Kit for Multiplex Immunohistochemistry (Akoya Biosciences, Marlborough, MA). After mIF staining, slides were scanned using the Vectra^®^ 3.0 Automated Quantitative Pathology Imaging System (PerkinElmer Inc., Hopkinton, MA). Quantitative assessment of cell markers was performed using inForm^®^ software (version 2.2.1, PerkinElmer Inc., Hopkinton, MA). Cell segmentation and phenotyping algorithms were reviewed and confirmed by study pathologists.

### Viral plaque assay and vaccinia virus neutralization assay

Viral plaque assays were performed on body fluid samples (blood, sputum, urine, pleural fluid) collected from patients immediately before and 24, 48, 72, and 96 hours after Olvi-vec treatment to assess for the presence of viral particles. Post-treatment tumor biopsies collected 2-5 days after treatment also underwent assessment for viral particles using viral plaque assays. In brief, patient samples were plated on confluent layers of CV-1 cells. Evaluation of virus infection was done by visual assessment of viral plaque in wells with both CV-1 cells and patient samples. Additionally, post-treatment serum samples obtained from patients 60 days after Olvi-vec treatment were assessed for the presence of Olvi-vec neutralizing antibodies *via* standard vaccinia virus neutralization assay and compared to corresponding pre-treatment serum samples.

### Effusion and pleural biopsy analysis

Pleural fluid and serum samples were obtained from patients both pre-treatment (baseline) and post-treatment (at 24, 48, 72, and 96 hours, and on days 2, 3, and 60). All specimens available were assessed for a panel of effector and suppressive cytokines using a 41-plex MILLIPLEX^®^ MAP Human Cytokine/Chemokine kit (MilliporeSigma, Burlington, MA). The kit was run on a Luminex^®^ 100/200™ System (Luminex Corporation, Austin, TX).

Values represent the mean of the duplicate wells ± standard deviation. These data were analyzed using IS 2.3 software (Luminex Software, Inc., Riverside, CA), Microsoft Excel and GraphPad Prism. Additionally, RNA isolation was performed on FFPE sections of tumor biopsies taken pre- and post-treatment using the RNeasy^®^ FFPE Kit in accordance with the manufacturer’s protocol (Qiagen, Germantown, MD). RNA concentration and purity were measured using the NanoDrop™ 2000/2000c Spectrophotometer (Thermo Scientific, Waltham, MA). RNA profiling was performed using the nCounter^®^ PanCancer Immune Profiling Panel by NanoString Technologies, Inc. (Seattle, WA).

### Statistical analyses

The sample size was based on a standard dose-escalation design. All statistical tests were two-sided, and statistical significance was defined as *p*<0.05. Data with normal distribution was assessed using paired t-test. Data without normal distribution was assessed using Wilcoxon matched-pairs signed-rank test. Analyses were conducted using R 4.1.2 (R Foundation for Statistical Computing, Vienna, Austria).

## Results

### Patient characteristics

From February 2013 to April 2015, 18 patients were enrolled who were treated in a dose-escalating fashion (**Table 1**). Fifteen patients had MPM (13 epithelioid, 1 biphasic, and 1 sarcomatoid), 2 had non-small cell lung cancer (1 adenocarcinoma and 1 squamous cell carcinoma), and 1 had triple negative breast cancer. 5 out of 18 patients were female, and the mean age of all patients was 66 years. All 3 patients with NSCLC or breast cancer and 1 patient with MPM had received previous lines of therapy.

**Table 1:** Clinical characteristics of patients treated in the phase I trial.

Patient ID	Age	Sex	Diagnosis	Histologic subtype	Previous regimens	Cohort	Dose (PFU)	# of doses
1	M	78	MPM	Epithelioid	None	1	1.00E+07	1
2	M	59	NSCLC	SCC	Chemotherapy	1	1.00E+07	1
3	M	73	MPM	Epithelioid	None	1	1.00E+07	1
4	M	54	MPM	Epithelioid	None	1	1.00E+07	1
5	M	62	MPM	Epithelioid	None	2	1.00E+08	1
6	M	74	MPM	Epithelioid	None	2	1.00E+08	1
7	M	74	NSCLC	ADC	Chemoradiotherapy	2	1.00E+08	1
8	F	63	MPM	Epithelioid	None	3	1.00E+09	1
9	M	81	MPM	Epithelioid	None	3	1.00E+09	1
10	M	76	MPM	Epithelioid	Chemotherapy	3	1.00E+09	1
11	F	51	MPM	Sarcomatoid	None	4	3.00E+09	1
12	F	43	TNBC		Neoadjuvant chemotherapy, surgery, adjuvant hormone therapy	4	3.00E+09	1
13	M	67	MPM	Biphasic	None	4	3.00E+09	1
14	M	74	MPM	Epithelioid	None	4 (expansion)	3.00E+09	1
15	M	70	MPM	Epithelioid	None	5	3.00E+09	3
16	F	67	MPM	Epithelioid	None	5	3.00E+09	3
17	M	79	MPM	Epithelioid	None	5	3.00E+09	3
18	F	47	MPM	Epithelioid	None	6	6.00E+09	3

ADC, adenocarcinoma; MPM, malignant pleural mesothelioma; NSCLC, non-small cell lung cancer; PFU, plaque-forming units; SCC, squamous cell carcinoma; TNBC, triple negative breast cancer.

### Feasibility and safety

**Figure 1:**
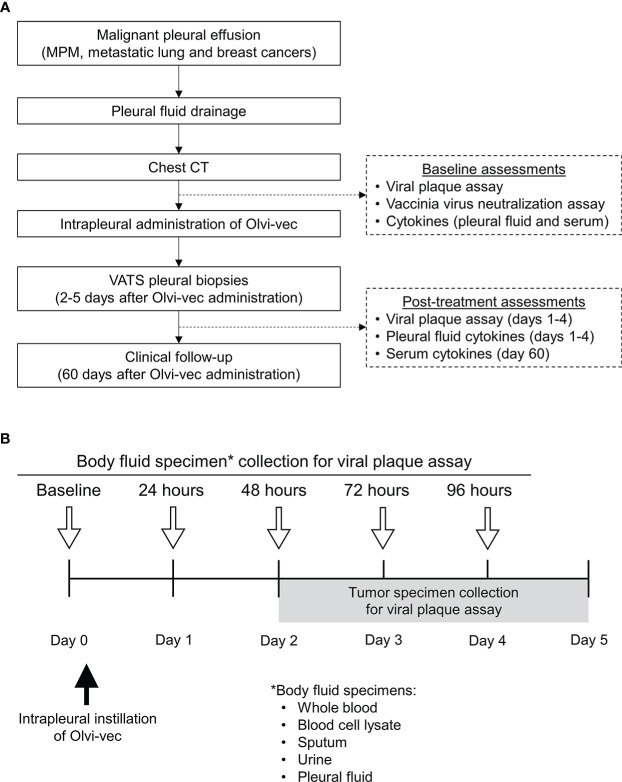
Protocol schema **(A)** and specimen collection schema for viral plaque assay **(B)**. *List of specimens collected.

Attenuated vaccinia virus (Olvi-vec) was administered intrapleurally as a bolus through a pleural catheter after complete evacuation of pleural effusion in all patients (**Figure 1**). The intrapleural administration of vaccinia virus was feasible, and there were no failures in administration of the agent. **Table 2** lists the adverse events that occurred at any grade (1–4) in ≥15% of the total cohort (n=18), up to day 60 post-treatment.

**Table 2:** Adverse events that occurred during the phase I trial (n=18).

Adverse event*	Any grade (%)	Grade 3 (%)	Grade 4 (%)
Hyperglycemia	18 (100%)	1 (6%)	0 (0%)
Anemia	17 (94%)	1 (6%)	0 (0%)
Hypocalcemia	13 (72%)	0 (0%)	1 (6%)
Hypoalbuminemia	13 (72%)	0 (0%)	0 (0%)
Pain	12 (67%)	0 (0%)	0 (0%)
Fatigue	11 (61%)	2 (11%)	0 (0%)
Fever	8 (44%)	0 (0%)	0 (0%)
Elevated ALT	7 (39%)	1 (6%)	0 (0%)
Nausea	7 (39%)	0 (0%)	0 (0%)
Chills	7 (39%)	0 (0%)	0 (0%)
General disorders and administration site conditions	6 (33%)	0 (0%)	0 (0%)
Hypophosphatemia	5 (28%)	2 (11%)	0 (0%)
Dyspnea	5 (28%)	1 (6%)	0 (0%)
Sinus tachycardia	5 (28%)	0 (0%)	0 (0%)
Hyperkalemia	5 (28%)	0 (0%)	0 (0%)
Elevated alkaline phosphate	5 (28%)	0 (0%)	0 (0%)
Flu-like symptoms	5 (28%)	0 (0%)	0 (0%)
Headache	5 (28%)	0 (0%)	0 (0%)
Elevated AST	4 (22%)	1 (6%)	0 (0%)
Thrombocytopenia	4 (22%)	0 (0%)	0 (0%)
Hyponatremia	4 (22%)	0 (0%)	0 (0%)
Elevated INR	4 (22%)	0 (0%)	0 (0%)
Lymphopenia	3 (17%)	3 (17%)	0 (0%)
Leukopenia	3 (17%)	0 (0%)	0 (0%)
Myalgia	3 (17%)	0 (0%)	0 (0%)
Generalized muscle weakness	3 (17%)	0 (0%)	0 (0%)
Diarrhea	3 (17%)	0 (0%)	0 (0%)

*Shown are adverse events that occurred in 15% or more of the study population up to day 60 post-treatment.

ALT, alanine aminotransferase; AST, aspartate aminotransferase; INR, international normalized ratio.

There was 1 reversible grade-4 laboratory abnormality (hypocalcemia). The most frequent grade 3 adverse events were lymphopenia (3 patients, 17%), fatigue (2 patients, 11%), and hypophosphatemia (2 patients, 11%). The most frequent grade 2 adverse events were anemia (5 patients, 28%), hyperglycemia (5 patients, 28%), and fever (4 patients, 22%). There were no dose-limiting toxicities, and maximally tolerated dose was not reached. As a result, the primary objective of establishing a recommended dose was not reached.

### Vaccinia detection and qualitative assessment of treatment effect in tumor

**Figure 2:**
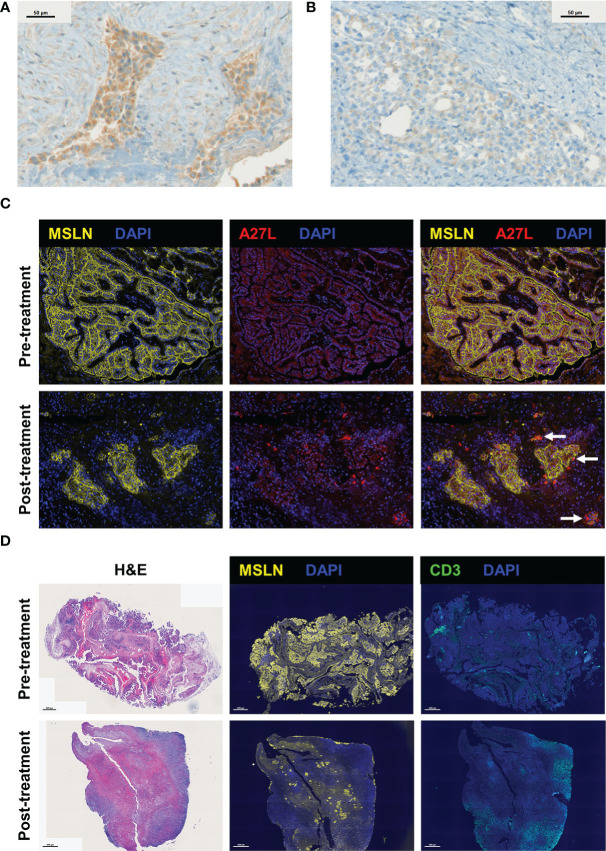
Visualization of Olvi-vec within tumor cells and the immune cell infiltrate in malignant pleural mesothelioma (MPM) tumors following Olvi-vec treatment. **(A)** Representative image of an immunohistochemistry (IHC)-stained section of a post-treatment tumor specimen from Patient #1 showing cytoplasmic positivity for Olvi-vec. **(B)** Representative image of an IHC-stained section from Patient #1 nine months later, showing weak/absent Olvi-vec staining. **(C)** Representative multiplex immunofluorescence (mIF) images of pre- and post-treatment tumor specimens from Patient #16, stained with anti-mesothelin and A27L (anti-Olvi-vec) antibodies. Arrows indicate tumor cells with cytoplasmic positivity for Olvi-vec, which are observed in the post-treatment specimen but not the pre-treatment specimen. **(D)** Representative images of pre- and post-treatment tumor specimens from Patient #16 stained with hematoxylin and eosin (H&E) (left panel), anti-mesothelin (MSLN) antibody (middle panel), and anti-CD3 antibody (right panel) in three consecutive cut sections. In the post-treatment specimen compared to pre-treatment, the density of tumor cells positive for MSLN is observed to be lower, and the density of immune cells positive for CD3 is observed to be higher.

Resected post-treatment samples from 14 patients were stained by immunohistochemistry with an antibody against Olvi-vec (A27L). Positive cytoplasmic expression was observed in 7 of 14 specimens (representative images shown in **Figures 2A, B**). When the A27L antibody was tested in a multiplex immunofluorescence panel with anti-mesothelin antibody on pre- and post-treatment specimens from Patient #16, cytoplasmic expression of Olvi-vec was observed in the post-treatment specimen but not the pre-treatment specimen (representative images shown in **Figure 2C**).

Resected samples from 13 patients were stained by multiplex immunofluorescence with a panel of antibodies against mesothelin (MSLN), CD3, CD4, CD8, and FoxP3. 4 patients had matched pre- and post-treatment specimens, and 9 patients had post-treatment specimens only. The density of MSLN+ tumor cells was qualitatively observed to decrease and the density of CD3+ immune cells was qualitatively observed to increase from pre-treatment to post-treatment specimens (representative images shown in **Figure 2D**).

### Quantitative assessment of treatment effect in tumor

**Figure 3:**
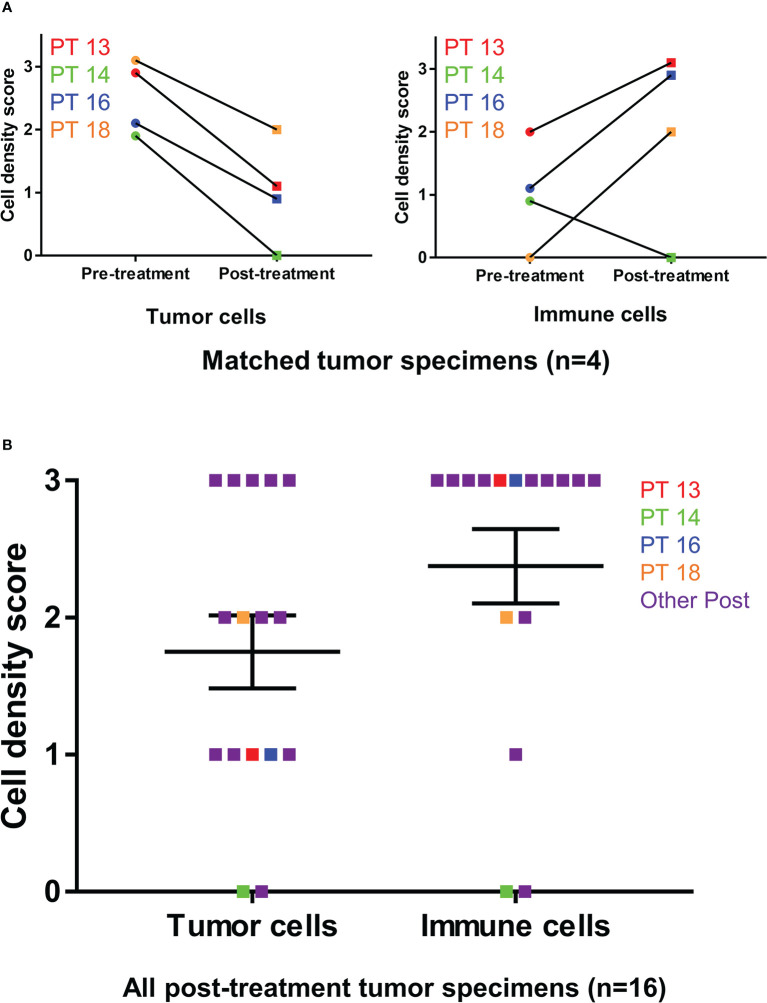
Tumor cell and immune cell density scoring before and after Olvi-vec therapy. **(A)** Matched MPM tumor sections obtained before and after Olvi-vec therapy underwent staining with hematoxylin and eosin and were scored for tumor cell density and immune cell density. **(B)** Tumor cell density scores and immune cell density were scored for all post-treatment tumor sections. Bars indicate mean and standard error of the mean (SEM).

Resected samples from 16 patients were stained with hematoxylin and eosin and independently scored semi-quantitatively for tumor-cell density and immune-cell density by two pathologists (**Table 3**). Four patients had matched pre- and post-treatment specimens, and 12 patients had post-treatment specimens only. When matched tumor specimens were compared (n=4), tumor cell density score decreased from pre-treatment to post-treatment in all patients (**Figure 3**). Immune cell density score increased from pre-treatment to post-treatment in 3 of 4 patients. Among all post-treatment tumor specimens (n=16), the average score per high power field (1 mm^2^) was lower for tumor cell density compared to immune cell density.

**Table 3:** Tumor and immune cell scoring of tumor specimens stained with hematoxylin and eosin.

Patient	Pathology	Cohort	Dose (PFU)	# of doses	Pre-treatment specimen		Post-treatment specimen	
					Tumor cells	Immune cells	Tumor cells	Immune cells
1	MPM (epithelioid)	1	1.00E+07	1	–	–	1	3
2	NSCLC (SCC)	1	1.00E+07	1	–	–	1	1
3	MPM (epithelioid)	1	1.00E+07	1	–	–	3	3
4	MPM (epithelioid)	1	1.00E+07	1	–	–	0	3
5	MPM (epithelioid)	2	1.00E+08	1	–	–	2	2
6	MPM (epithelioid)	2	1.00E+08	1	–	–	3	3
7	NSCLC (ADC)	2	1.00E+08	1	–	–	–	–
8	MPM (epithelioid)	3	1.00E+09	1	–	–	–	–
9	MPM (epithelioid)	3	1.00E+09	1	–	–	3	3
10	MPM (epithelioid)	3	1.00E+09	1	–	–	2	3
11	MPM (sarcomatoid)	4	3.00E+09	1	–	–	3	3
12	TNBC	4	3.00E+09	1	–	–	2	0
13	MPM (biphasic)	4	3.00E+09	1	3	2	1	3
14	MPM (epithelioid)	4 (expansion)	3.00E+09	1	2	1	0	0
15	MPM (epithelioid)	5	3.00E+09	3	–	–	3	3
16	MPM (epithelioid)	5	3.00E+09	3	2	1	1	3
17	MPM (epithelioid)	5	3.00E+09	3	–	–	1	3
18	MPM (epithelioid)	6	6.00E+09	3	3	0	2	2

– Insufficient sample

ADC, adenocarcinoma; MPM, malignant pleural mesothelioma; NSCLC, non-small cell lung cancer; PFU, plaque-forming units; SCC, squamous cell carcinoma; TNBC, triple negative breast cancer.

**Figure 4:**
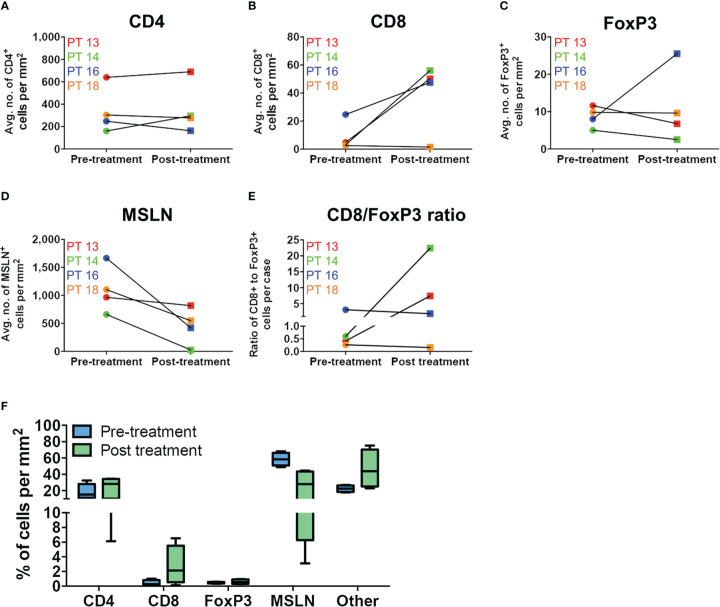
Immune cell infiltration in matched MPM tumor specimens before and after Olvi-vec therapy. **(A–D)** Matched MPM tumor sections obtained before and after Olvi-vec therapy underwent mIF staining and quantification of **(A)** CD4+ T cells, **(B)** CD8+ T cells, **(C)** FoxP3+ T cells, and **(D)** mesothelin (MSLN)+ tumor cells. **(E)** The ratio of CD8+ to FoxP3+ T cells in pre- and post-treatment tumor sections was calculated to identify patients with immunogenic (CD8+/FoxP3+ >1) vs. immune suppressive (CD8+/FoxP3+ <1) tumor microenvironments. **(F)** Immune cell populations present in matched pre- and post-treatment tumor sections were expressed as a percentage of total cells per mm^2^. “Other” cells are DAPI+ but negative for CD4, CD8, FoxP3, and MSLN—they may be fibroblasts, mesothelial cells, or other classes of immune cells. Bars indicate mean, interquartile range, and SEM.

**Figure 5:**
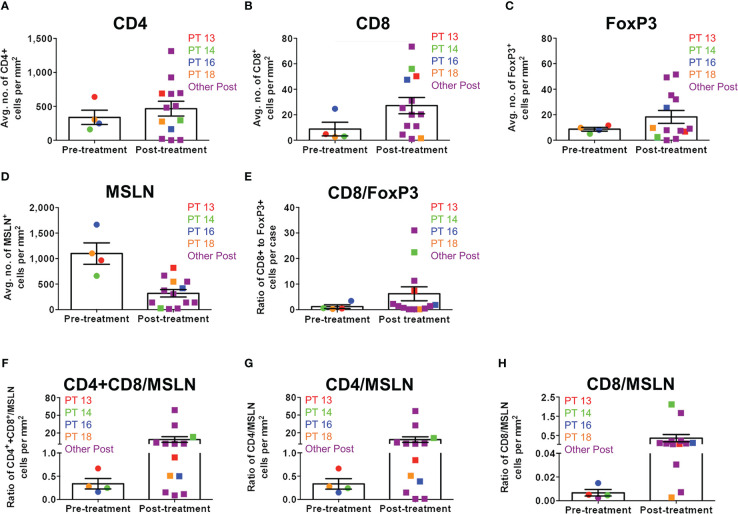
Immune cell infiltration ratios in all MPM tumor specimens before and after Olvi-vec therapy. **(A–D)** MPM tumor sections obtained before and after Olvi-vec therapy underwent multiplex immunofluorescence (mIF) staining and quantification of **(A)** CD4+ T cells, **(B)** CD8+ T cells, **(C)** FoxP3+ T cells, and **(D)** mesothelin (MSLN)+ tumor cells. **(E)** The ratio of CD8+ to FoxP3+ T cells (high ratio indicating relative immunogenicity, low ratio indicating relative immune suppression) in pre- and post-treatment tumor sections was calculated. **(F–H)** Ratios of **(F)** total T cells (CD4+ and CD8+) to MSLN+ tumor cells, **(G)** CD4+ T cells to MSLN+ tumor cells, and **(H)** CD8+ T cells to MSLN+ tumor cells were calculated. Bars indicate mean and SEM.

When matched tumor specimens were stained using multiplex immunofluorescence and then compared (n=4), mean CD8+ cells per mm^2^ increased in 3 of 4 patients (**Figure 4**). Mean MSLN+ tumor cells per mm^2^ decreased from pre-treatment to post-treatment in all patients. Comparing available pre-treatment specimens to all post-treatment specimens (n=13), mean CD8+ cells increased and mean MSLN+ tumor cells decreased, although neither achieved statistical significance (**Figure 5**).

**Figure 6:**
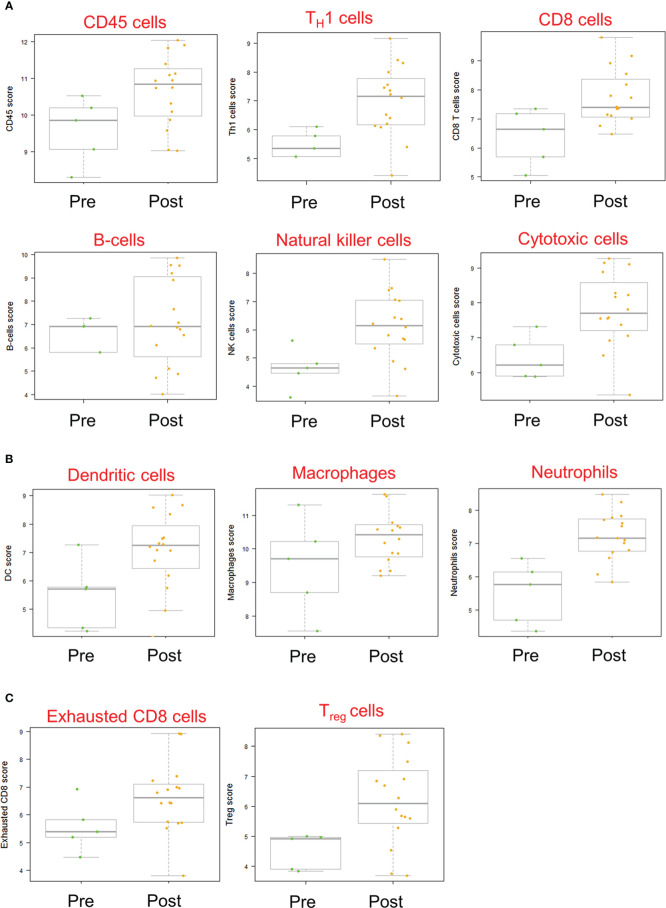
Immune cell scores based on mRNA transcript abundance before and after Olvi-vec therapy. **(A–C)** Total RNA was isolated from MPM tumor sections before and after Olvi-vec therapy. The number of mRNA transcripts specific to **(A)** lymphoid, **(B)** myeloid, and **(C)** exhausted and regulatory cell types were quantified. A score increase of one indicates a two-fold increase in cell population abundance in a sample. Bars indicate mean, interquartile range, and range.

**Figure 7:**
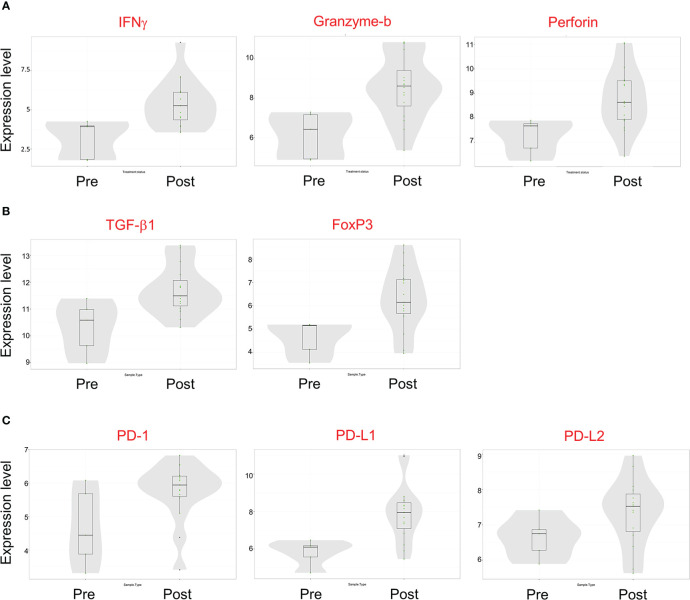
mRNA transcript expression of immune-modulatory proteins before and after Olvi-vec therapy. **(A–C)** Total RNA was isolated from MPM tumor sections before and after Olvi-vec therapy. The number of mRNA transcripts for **(A)** immune effector proteins, **(B)** immune suppressive proteins, and **(C)** immune checkpoint regulatory proteins were quantified. Bars indicate mean, interquartile range, and range.

Gene expression analysis of pre- and post-treatment tumor specimens (n=16), using nCounter immune cell type scoring module, revealed increased scores (i.e., change in score by 1 unit, indicating twice the abundance of that cell type) for CD45+, Th1+, Tregs, CD8+, exhausted CD8+, NK+, cytotoxic cells, dendritic cells, macrophage, and neutrophil immune cell populations in post-treatment tumor specimens compared to pre-treatment specimens (**Figure 6**). Similarly, scoring of individual protein mRNA levels pre- and post-treatment tumor specimens (n=13) revealed increased scores in immune effector proteins (IFN-γ, granzyme B, perforin), immune suppressive proteins (TGFβ, FoxP3), and immune checkpoint regulatory proteins (PD-1, PD-L1, PD-L2) following treatment (**Figure 7**). The data discussed in this publication have been deposited in NCBI’s Gene Expression Omnibus (31) and are accessible through GEO Series accession number GSE223395 (https://www.ncbi.nlm.nih.gov/geo/query/acc.cgi?acc=GSE223395).

### Vaccinia detection in body fluids and anti-vaccinia immune response

Vaccinia virus was detectable by viral plaque assay in the pleural fluid of 7 patients (**Table 4A**). Vaccinia was initially detected in pleural fluid at the dose 1.00E8 PFU (Cohort 2) and exhibited dose-dependent increase in PFU/mL in Cohorts 3, 4, 5, and 6. Viral plaque assay was also positive for vaccinia in the post-treatment tumor lysate of 4 patients. There was minimal viral shedding into other compartments, as shown by low positivity in the urine of 2 patients, blood lysate of 1 patient, and sputum of 1 patient (**Table 4B**). Of note, among patients who received multiple doses of Olvi-vec (Cohorts 5 and 6), significant increase in the number of plaque-forming units was observed in the pleural fluid of 2 patients.

**Table 4A:** Results of viral plaque assay (pleural fluid).

Patient	Pathology	Dose (PFU)	# of doses	Olvi-vec plaque-forming units/mL (dilution factor)					
				Pleural fluid					Post-treatment tumor lysate
				Baseline	24 hours	48 hours	72 hours	96 hours	
1	MPM (epithelioid)	1.00E+07	1	0	0	0			4
2	NSCLC (SCC)	1.00E+07	1	0	0	0			0
3	MPM (epithelioid)	1.00E+07	1	0	0	0			0
4	MPM (epithelioid)	1.00E+07	1	0	0	0			0
5	MPM (epithelioid)	1.00E+08	1	0	0	0			0
6	MPM (epithelioid)	1.00E+08	1	0	–	0			0
7	NSCLC (ADC)	1.00E+08	1	0	0	0	–	7	–
8	MPM (epithelioid)	1.00E+09	1	0	42	60 (10)		51	18 (100)
9	MPM (epithelioid)	1.00E+09	1	–	0	0			0
10	MPM (epithelioid)	1.00E+09	1	0	–	0		0	0
11	MPM (sarcomatoid)	3.00E+09	1	0	44 (100)	126 (10)		221	0
12	TNBC	3.00E+09	1	0	0	0			0
13	MPM (biphasic)	3.00E+09	1	0	0	0			0
14	MPM (epithelioid)	3.00E+09	1	0	5 (10)	0			60
15	MPM (epithelioid)	3.00E+09	3	0	339	129 (10)	399 (10)	48	0
16	MPM (epithelioid)	3.00E+09	3	0	6 (1000)	33 (1000)	72 (1000)	30 (1000)	56 (1000)
17	MPM (epithelioid)	3.00E+09	3	0	0	0	26	0	0
18	MPM (epithelioid)	6.00E+09	3	0	3	4	32	9	0

-Insufficient sample ⬛Not performed ADC, adenocarcinoma; MPM, malignant pleural mesothelioma; NSCLC, non-small cell lung cancer; PFU, plaque-forming units; SCC, squamous cell carcinoma; TNBC, triple negative breast cancer.

**Table 4B:** Results of viral plaque assay (other specimens).

Patient	Pathology	Dose (PFU)	# of doses	Specimen type	Olvi-vec plaque-forming units/mL (dilution factor)				
					Specimen				
					Baseline	24 hours	48 hours	72 hours	96 hours
1	MPM (epithelioid)	1.00E+07	1	Urine	0	0	14		
3	MPM (epithelioid)	1.00E+07	1	Urine	4	0	0		
5	MPM (epithelioid)	1.00E+08	1	Blood lysate	0	2	0		
18	MPM (epithelioid)	6.00E+09	3	Sputum	7	0	0	17	0

⬛Not performed

MPM, malignant pleural mesothelioma; PFU, plaque-forming units.

Among 7 patients whose baseline serum was available to perform vaccinia virus neutralization assay, 4 patients had low levels of anti-vaccinia neutralizing antibodies pre-treatment, and 3 had no neutralizing antibodies (**Table 5**). Five of the patients had high levels of neutralizing antibodies at day 60 post-treatment.

**Table 5:** Results of vaccinia virus neutralization assay (serum).

Patient	Pathology	Dose (PFU)	# of doses	Specimen type	Vaccinia Virus Neutralization Assay (dilution factor)	
					Baseline	Day 60
1	MPM (epithelioid)	1.00E+07	1	Serum	–	–
2	NSCLC (SCC)	1.00E+07	1	Serum	–	–
3	MPM (epithelioid)	1.00E+07	1	Serum	–	–
4	MPM (epithelioid)	1.00E+07	1	Serum	–	–
5	MPM (epithelioid)	1.00E+08	1	Serum	–	–
6	MPM (epithelioid)	1.00E+08	1	Serum	–	–
7	NSCLC (ADC)	1.00E+08	1	Serum	–	–
8	MPM (epithelioid)	1.00E+09	1	Serum	–	–
9	MPM (epithelioid)	1.00E+09	1	Serum	–	–
10	MPM (epithelioid)	1.00E+09	1	Serum	–	–
11	MPM (sarcomatoid)	3.00E+09	1	Serum	Negative	–
12	TNBC	3.00E+09	1	Serum	Negative	–
13	MPM (biphasic)	3.00E+09	1	Serum	Positive (20)	Positive (2560)
14	MPM (epithelioid)	3.00E+09	1	Serum	Positive (10)	Positive (640)
15	MPM (epithelioid)	3.00E+09	3	Serum	Positive (10)	Positive (2560)
16	MPM (epithelioid)	3.00E+09	3	Serum	Negative	Positive (1280)
17	MPM (epithelioid)	3.00E+09	3	Serum	Positive (40)	Positive (20480)
18	MPM (epithelioid)	6.00E+09	3	Serum	–	–

–Insufficient sample

ADC, adenocarcinoma; MPM, malignant pleural mesothelioma; NSCLC, non-small cell lung cancer; PFU, plaque-forming units; SCC, squamous cell carcinoma; TNBC, triple negative breast cancer.

### Pleural fluid and serum cytokine analysis

**Figure 8:**
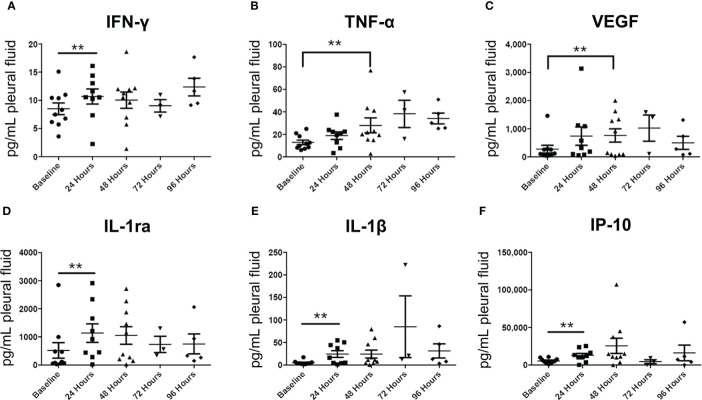
Pleural fluid cytokine profiles before and after Olvi-vec therapy. **(A–F)** Pleural fluid sampled before and after Olvi-vec therapy (at 24 hours, 48 hours, 72 hours, and 96 hours post-treatment) was evaluated for concentrations of cytokines. Bars indicate mean and SEM. **p<0.05, paired t-test.

**Figure 9:**
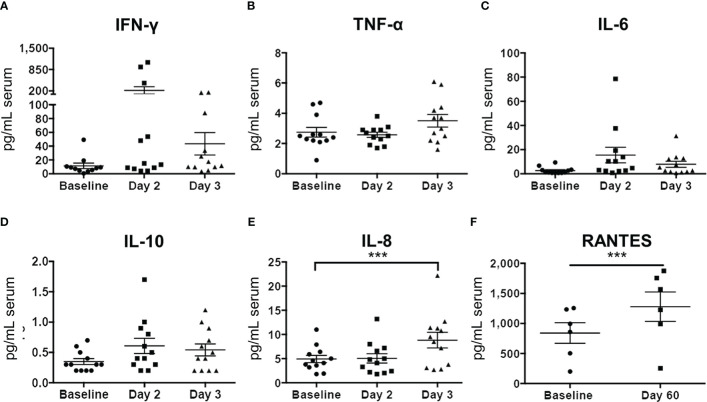
Serum cytokine profiles before and after Olvi-vec therapy. **(A–F)** Serum sampled before and after Olvi-vec therapy (at Day 2, Day 3, and Day 60) was evaluated for concentrations of cytokines. Bars indicate mean and SEM. ***p<0.01, paired t-test.

Luminex analysis of pleural fluid specimens from baseline to up to 96 hours following treatment indicated significant increase in the levels of the following cytokines by 48 hours: IFN-γ, TNF-α, VEGF, IL-1ra, IL-1β, and IP-10 (**Figure 8**). In contrast, analysis of serum specimens showed an increase only in IL-8 levels from baseline to day 3 following treatment (*p*=0.0065; **Figure 8**). By day 60, only RANTES (CCL5) was found to be significantly elevated in serum compared to baseline (*p*=0.0276; **Figure 9**).

### Long-term outcomes

**Figure 10:**
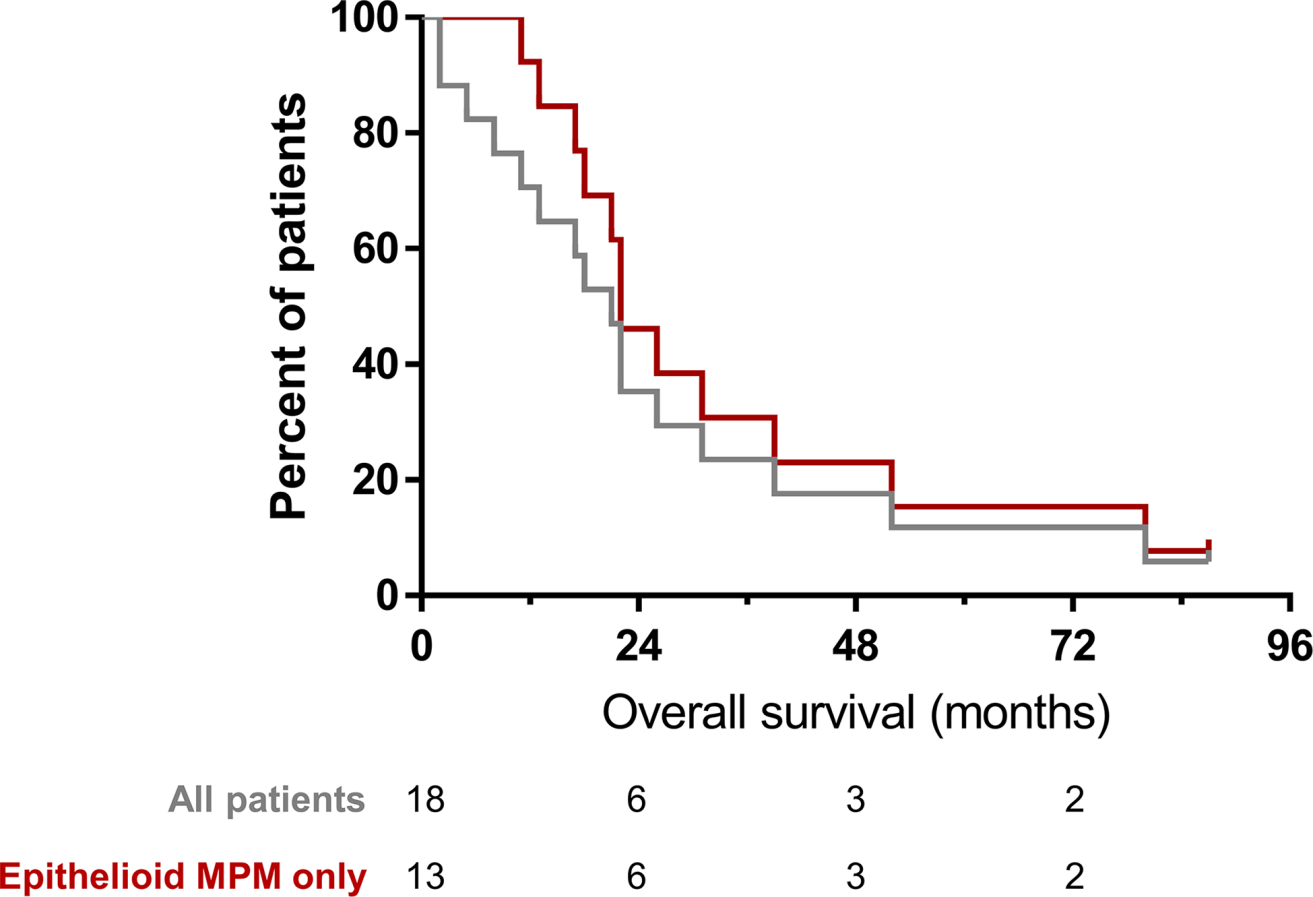
Overall survival of patients following Olvi-vec therapy.

All patients received subsequent other therapies as determined by treating physician following participation in the trial. Among all patients, median overall survival (OS) was 19.5 months. The median OS among patients who had MPM was 22 months (**Figure 10**). One patient with epithelioid MPM is alive; 87 months after treatment, the patient received other treatments that are standard of care for patients with MPM.

## Discussion

Our phase I study of intrapleural oncolytic viral therapy is based on strong rationale developed in preclinical models of malignant pleural mesothelioma (32–34). The strength of our phase I study is the correlative analysis performed on pre- and post-treatment pleural effusions and pleural biopsies along with systemic immune response assessment by cytokine analysis following intrapleural oncolytic viral therapy. Intrapleural administration of Olvi-vec treatment is feasible, safe, and associated with induction of effector immune responses. All patients received treatment with the established dose *via* intrapleural delivery. Olvi-vec was detected using direct and indirect methods in resected tumor specimens and pleural fluid collected post-treatment. The treatment was safe, with one grade 4 laboratory abnormality and no treatment-associated mortalities noted. There were no dose-limiting toxicities or dose de-escalations, and the maximally tolerated dose was not reached. Therefore, a recommended dose was not established. Presence of the vaccinia virus within tumor cells was detectable 2-5 days after treatment and associated with local reduction in tumor cell density and an increase in immune cell density. Specifically, CD8+ T-cell density increased, indicating the generation of treatment-induced immunogenicity. Gene expression analysis showed increases in multiple immune cell populations (including CD8+, CD45+, Th1+, Tregs, NK cells, macrophages, neutrophils, dendritic cells, and cytotoxic cells), as well as increased concentration of effector proteins, immune checkpoint proteins, and cytokines in post-treatment tumor and pleural fluid samples. Viral shedding outside the pleural compartment was observed in only 4 patients. The number of plaque-forming units in pleural fluid was significantly increased in 2 patients who received multiple doses of Olvi-vec (cohorts 5 and 6). Most importantly, there was minimal systemic immune activation following intrapleural treatment, with only 2 cytokines noted to be elevated in serum. Among trial patients who had MPM, median OS was 22 months following treatment.

Our observations in treating patients with MPE-associated immunosuppressive microenvironment are similar to published studies of vaccinia viral therapy without immunosuppression at the administered site. Administered as systemic therapy in clinical trials, vaccinia viral therapy was associated with a trend toward improved progression-free survival and increased CD4+/Treg ratio in patients with metastatic breast cancer (35). In patients with advanced colorectal liver metastases and metastatic melanoma, vaccinia viral therapy was associated with significant increases in IFN-stimulated and pro-inflammatory cytokines, as well as NK-cell activation and CD8+ proliferation (36). Vaccinia viral therapy had no observed benefit in patients with advanced soft tissue sarcoma (37).

Intrapleural oncolytic viral therapy has been investigated by use of multiple oncolytic viruses. In patients with MPM, intrapleural delivery of adenoviral gene-mediated cytotoxic therapy has been investigated. Adenoviral vectors have been used for gene transfer of cytokines [such as IFN-α (8) and IFN-β (38)] and enzymes (such as TK) that potentiate cytotoxic activity of subsequently administered ganciclovir (39) or valacyclovir (7). These trials reported isolated instances of grade 4 pericardial tamponade (38), grade 4 hypotension (7), and severe flu-like symptoms requiring dose de-escalation (8). We did not observe any dose-limiting or treatment-related grade ≥4 toxicities in our trial. Adenovirus-mediated IFN-β and IFN-α gene transfer were reported to be associated with increases in activated NK-cell populations (8, 40). Our correlative data noted a 2-fold increase in NK cells based on gene expression analysis. Adenovirus-mediated TK and IFN gene transfers were associated with survival greater than 2 years in multiple patients (7, 8, 40, 41), as was observed following vaccinia treatment in our study. However, the survival observed cannot be attributed to the treatment agent due to the phase I nature of the study and limited clinical anti-tumor efficacy observed immediately after the treatment.

Following adenovirus-mediated TK/ganciclovir therapy, gene transfer was observed to be limited to superficial layers of the tumor (39). Yet, radiologic response and persistent clinical response were observed in 3 patients (41), prompting the authors to hypothesize that the therapy had immune activating effects in addition to direct cytotoxicity. Indeed, administration of adenovirus TK has been shown to increase PD-L1 expression on tumor cells (7). In the current trial we observed increased expression of PD-1, PD-L1, and PD-L2 mRNA transcripts following vaccinia virus treatment. We also observed presence of vaccinia virus in the tumor as identified by multiplex immunofluorescence along with associated immune activation signature by nanostring and cytokine analyses. Oncolytic virus-induced tumor cytotoxicity may potentially shift the balance of the immune microenvironment towards activation through pathogen-associated and damage-associated molecular pattern signaling. These observations provide rationale to the addition of checkpoint blockade to vaccinia virus treatment (42) to enhance immune activation and antitumor efficacy. In preclinical studies, vaccinia virus combined with anti-PD1 therapy caused tumor reduction in glioblastoma (42), and vaccinia virus combined with MEK inhibitory therapy resulted in enhanced cytotoxicity in doxorubicin-resistant ovarian cancer (43).

In addition to effector immune responses (an increase in CD45+, Th1+, CD8+, NK+, cytotoxic T cells, and dendritic cells), we also observed an increase in exhausted CD8+ T cells and macrophages indicating the suppressor immune response. However, it is not certain whether these alterations are limited to tumors with pre-existing immune suppressor responses that are augmented following vaccinia viral therapy. In addition, our clinical trial was limited by the inclusion of a small number of patients from a single institution, who had heterogeneous types and stages of disease. Not all specimens investigated were available from all patients, a limitation inherent in a phase I clinical trial. Nevertheless, our correlative analyses demonstrating immune activation support the potential utility of vaccinia virus as an intrapleural oncolytic treatment for patients with MPM.

## Data availability statement

The data discussed in this publication have been deposited in NCBI’s Gene Expression Omnibus (31) and are accessible through GEO Series accession number GSE223395 (https://www.ncbi.nlm.nih.gov/geo/query/acc.cgi?acc=GSE223395).

## Ethics statement

The studies involving human participants were reviewed and approved by the Memorial Sloan Kettering Cancer Center Institutional Review Board (IRB# 12-169). The patients/participants provided their written informed consent to participate in this study.

## Author contributions

NC: Data curation, Formal analysis, Investigation, Methodology, Software, Validation, Visualization, Roles/Writing – original draft, Writing – review & editing; JC: Data curation, Formal analysis, Investigation, Methodology, Software, Validation, Visualization, Roles/Writing – original draft, Writing – review & editing; EM: Data curation, Formal analysis, Investigation, Methodology, Writing – review & editing; RB: Data curation, Investigation, Writing – review & editing; JS: Data curation, Investigation, Writing – review & editing; SB: Data curation, Investigation, Writing – review & editing; AM: Data curation, Investigation, Writing – review & editing; MZ: Data curation, Investigation, Writing – review & editing; PA: Conceptualization, Data curation, Formal analysis, Funding acquisition, Investigation, Methodology, Project Administration, Resources, Software, Supervision, Validation, Visualization, Roles/Writing – original draft, Writing – review & editing; VR: Conceptualization, Data curation, Funding acquisition, Investigation, Resources, Supervision, Writing – review & editing. All authors contributed to the article and approved the submitted version.
