# Supplementary material for: Correlative analysis from a phase I clinical trial of intrapleural administration of oncolytic vaccinia virus (Olvi-vec) in patients with malignant pleural mesothelioma
Source: Front Immunol. 2023 Feb 16;14:1112960. doi: 10.3389/fimmu.2023.1112960 (PMC9977791; doi:10.3389/fimmu.2023.1112960)
Supplement: Supplementary file 1 [file Table_1.docx]

Supplementary Material

# Supplementary Tables

**Supplementary Table 1.** Antibodies used in multiplex immunofluorescence staining of pre- and post-treatment tumor specimens

| **Marker** | **Dilution** | **Clone** | **Company** |
| --- | --- | --- | --- |
| CD3 | 1:150 | SP7 | Abcam |
| CD4 | 1:1500 | EPR6855 | Abcam |
| CD8 | 1:2 | C8/144B | ThermoFisher |
| FoxP3 | 1:500 | 236A/E7 | Abcam |
| MSLN | 1:500 | MN-1 | Rockland |
| Olvi-vec | 1:2 | A27L | Abcam |
