# Supplementary material for: Correlative analysis from a phase I clinical trial of intrapleural administration of oncolytic vaccinia virus (Olvi-vec) in patients with malignant pleural mesothelioma
Source: Front Immunol. 2023 Feb 16;14:1112960. doi: 10.3389/fimmu.2023.1112960 (PMC9977791; doi:10.3389/fimmu.2023.1112960)
Supplement: Supplementary file 2 [file DataSheet_1.pdf]

MEMORIAL SLOAN-KETTERING CANCER CENTER
IRB PROTOCOL

IRB#: 12-169 A (8)

Phase I Study of Intra-pleural Administration of GL-ONC1, a Genetically Modified Vaccinia Virus, in Patients with Malignant Pleural Effusion: Primary, Metastases and Mesothelioma

PROTOCOL FACE PAGE FOR
MSKCC THERAPEUTIC/DIAGNOSTIC PROTOCOL

Principal Investigator/Department:	Valerie Rusch, MD	Surgery/Thoracic
Co-Principal Investigator(s)/Department:	Lee Krug, MD<br>Prasad Adusumilli, MD	Medicine/Thoracic<br>Surgery/Thoracic
Investigator(s)/Department:	Mark Kris, MD<br>Naiyer Rizvi, MD<br>Gregory Riely, MD, PhD<br>Maria C. Pietanza, MD<br>Marjorie Zauderer, MD<br>Paul K. Paik, MD<br>Jamie E. Chaft, MD<br>Charles Rudin, MD<br>Alexander Drilon, MD<br>Helena Yu, MD<br>Nancy Kemeny, MD<br>Jacqueline Bromberg, MD, PhD<br>Kent Sepkowitz, MD<br>Mark G. Klang, PhD<br>Michelle Ginsberg MD<br>Martin Fleisher, PhD<br>Camelia S. Sima MD, MS<br>Andre Luis Moreira, MD, PhD	Medicine/Thoracic<br>Medicine/Thoracic<br>Medicine/Thoracic<br>Medicine/Thoracic<br>Medicine/Thoracic<br>Medicine/Thoracic<br>Medicine/Thoracic<br>Medicine/Thoracic<br>Medicine/Thoracic<br>Medicine/Thoracic<br>Medicince/GI<br>Medicine/Breast<br>Medicine/Infectious Disease<br>SKI/Research Pharmacy<br>Radiology<br>Laboratory Medicine<br>Epidemiology & Biostatistics<br>Pathology
Consenting Professional(s)/Department:	Valerie Rusch, MD<br>Prasad Adusumilli, MD<br>Lee Krug, MD<br>Marjorie Zauderer, MD	Surgery/Thoracic<br>Surgery/Thoracic<br>Medicine/Thoracic<br>Medicine/Thoracic

Please Note: A Consenting Professional must have completed the mandatory Human Subjects Education and Certification Program.

Memorial Sloan-Kettering Cancer Center
1275 York Avenue
New York, New York 10065

Table of Contents

1.0 PROTOCOL SUMMARY AND/OR SCHEMA 3
2.0 OBJECTIVES AND SCIENTIFIC AIMS 7
3.0 BACKGROUND AND RATIONALE 7
4.0 OVERVIEW OF STUDY DESIGN/INTERVENTION 22
4.1 Design 22
4.2 Intervention 22
5.0 THERAPEUTIC/DIAGNOSTIC AGENTS 25
6.0 CRITERIA FOR SUBJECT ELIGIBILITY 30
6.1 Subject Inclusion Criteria 30
6.2 Subject Exclusion Criteria 30
7.0 RECRUITMENT PLAN 31
8.0 PRETREATMENT EVALUATION 32
9.0 TREATMENT/INTERVENTION PLAN 32
10.0 EVALUATION DURING TREATMENT/INTERVENTION 34
11.0 TOXICITIES/SIDE EFFECTS 36
12.0 CRITERIA FOR THERAPEUTIC RESPONSE/OUTCOME ASSESSMENT 36
13.0 CRITERIA FOR REMOVAL FROM STUDY 39
14.0 BIOSTATISTICS 40
15.0 RESEARCH PARTICIPANT REGISTRATION AND RANDOMIZATION PROCEDURES 41
15.1 Research Participant Registration 41
15.2 Randomization 41
16.0 DATA MANAGEMENT ISSUES 42
16.1 Quality Assurance 42
16.2 Data and Safety Monitoring 42
17.0 PROTECTION OF HUMAN SUBJECTS 44
17.1 Privacy 45
17.2 Serious Adverse Event (SAE) Reporting 45
17.2.1 46
18.0 INFORMED CONSENT PROCEDURES 49
19.0 REFERENCES 50
20.0 APPENDICES 51

1.0 PROTOCOL SUMMARY AND/OR SCHEMA

**Title of study**	
A Phase I Study of intra-pleural administration of GL-ONC1, a genetically modified vaccinia virus, in patients with malignant pleural effusion related either to malignant pleural mesothelioma or to metastatic disease.	
**First Study Centre**	
Study centre:	Memorial Sloan-Kettering Cancer Center
Principal Investigator:	Dr. Valerie Rusch
Co-Investigator:	Dr. Lee Krug<br>Dr. Prasad Adusumilli
**Protocol**	
Protocol number:	GL-ONC1-003/MSKCC
Phase of development:	Phase I

Objectives:
The primary objective is to determine a recommended dose of an attenuated vaccinia virus, GL-ONC1, when administered to patients with malignant pleural effusion (primary non-small-cell lung carcinoma, malignant mesothelioma, and other histologies).

Secondary objectives include the feasibility, safety and tolerability of intrapleural vaccinia virus; the detection of virus in body fluids; evaluation of viral appearance in tumor; and evaluation of anti-vaccinia virus immune response (e.g. antibody responses). Any evidence of anti-tumor activity will be noted.

Methodology:
Design:
This is an open-label, dose-escalating, non-randomized, single-center phase I study of GL-ONC1 administered intrapleurally as a single dose and now escalating to three consecutive daily doses in patients with a diagnosis (histologically or cytologically documented) of malignant pleural effusions. The total number of patients studied will depend on the number of dose levels tested up to a maximum dose of 1×10^{10} pfu or until Maximum Tolerated Dose (MTD) is reached. For this phase I study, a maximum number of 34 evaluable patients will be enrolled; this number includes 3 additional patients enrolled in an expansion cohort at the MTD.

Population:
Patients who have been diagnosed with malignant pleural effusion (MPE) from primary non-small-cell lung carcinoma, breast cancer, malignant mesothelioma and other histologies.

Dose Escalation Schema:
Patients will be enrolled in groups of three and individually assessed for safety and dose-limiting toxicity (DLT). The dose escalation scheme is shown below. Escalation/de-escalation decisions will be recommended by the MSKCC Data Safety Monitoring Committee (DSMC). The DSMC will make the final decision to proceed to the next dose level. The DSMC will review every major toxicity.

Table: Dose level by cohort (fixed dose independent of body weight)

Cohort	Dose **	Number of doses	Total volume of each injection
-1*	1×10^{6}	1	Final volume of preparation will be 500 mL, to be administered as a bolus.
+1	1×10^{7}	1	
+2	1×10^{8}	1	
+3	1×10^{9}	1	
+4	3×10^{9}	1	
+5***	$3 x 10^{9}$	3	
+6***	$6 x 10^{9}$	3	
+7***	$1 x 10^{10}$	3	
Phase IIa (expansion)	(selected dose)***	3	

* Necessary only if a DLT is encountered at the initial dose level.
**Dose de-escalation, if needed, will be done to intermediate levels that are one-half log lower, unless DSMC advises for full dose de-escalation
***Cohorts 5 or 6 or 7 may be selected as the expansion cohort based on safety. Three additional patients will be treated at the selected dose for expansion.

The first patient per cohort must be treated and complete 14 days of post-treatment evaluation prior to the treatment of the remaining two patients in that cohort. All patients treated in that cohort must be evaluated for 14 days, prior to treatment of the next dose cohort. However, the next patients can be pre-screened to be ready for the next enrollment.

Maximum Tolerated Dose (MTD):
If 1 patient out of 3 in a dose group experiences a Dose Limiting Toxicity (DLT) with a determination of relatedness to GL-ONC1 (i.e., possibly, probably, definitely), 3 more patients will be added to that dose group. If 2 or more patients out of a dose group of 6 patients experience any severe adverse reaction with a determination of relatedness to GL-ONC1 (i.e., possibly, probably, definitely), it is considered that a DLT for that cohort has been reached. Dose de-escalation will be to an intermediate level of one-half log lower dose, unless DSMC recommends de-escalation by one full-dose. If tolerated, that dose cohort will be the MTD. For cohorts 4 and 5, de-escalation to cohort 3 is a one-half log reduction. If de-escalation is required for either Cohort 6 or Cohort 7, dose reduction will be to the prior cohort dose, or to a dose level in between.

With or without identifying the MTD is identified, the recommended dose level will be expanded by 3 additional patients to a maximum of 6 total patients who will be treated to better define the tolerability, viral replication and pharmacodynamics of the dose level and schedule. If any DLTs are seen in this group of patients, the principal investigator or designee, in consultation with the DSMC, will discuss possible de-escalation of dose level.

Maximum Feasible Dose
If patients treated in cohort 7 tolerate the dose/dose regimen no further dose increase is planned. In such case the dose of $1 x 10^{10}$ pfu administered on 3 consecutive days would be considered as Maximum Feasible Dose.
Safety evaluations:
The safety of GL-ONC1 will be assessed by the evaluation of the type, frequency, and severity of adverse events (AEs), changes in clinical laboratory tests (hematological and chemistry), immunogenicity and physical examination. All AEs and laboratory toxicities will be graded on the CTCAE (version 4).

Single infusion (Cohorts +1 to +4)
At baseline (prior to treatment), daily during the first 3 days after treatment, and at termination of study (day 60±5), laboratory testing will consist of Complete Blood Count

(CBC), SMA-12 blood chemistries, and routine urinalysis.

Multiple infusions (Cohorts +5 to IIa)
At baseline (prior to treatment), on Days 2 & Day 3 (if applicable), and daily for 2 days after the last infusion, at the PO clinical follow-up day visit (between day 14 and 30), and at the termination of study (day 60±10), laboratory testing will consist of CBC, comprehensive metabolic panel, serum mesothelin related peptide (SMRP) & LDH tests. SMRP will be performed only in mesothelioma patients for whom it is considered a clinically billable serum marker test.

Single Infusion (Cohorts +1 to +4)
Vital signs (i.e., blood pressure, temperature, pulse rate) will be charted immediately before treatment and at 30 (+/-15) minutes, 60(+/-15) minutes and 120(+/-15) minutes, 8(+/-1) hours, and 16(+/-1) hours following virus treatment, every inpatient day thereafter, and at the Clinical Follow-up Visit (between Day 14 and 30), and at termination of study (day 60±5). An ECG will be performed at baseline.

Multiple infusions (Cohorts +5 to IIa)
Vital signs (i.e., blood pressure, temperature, pulse rate) will be charted before treatment and at 30 (+/-15) minutes, 60(+/-15) minutes and 120(+/-15) minutes, 8(+/-1) hours, and 16(+/-1) hours following each virus treatment and at least daily every inpatient day thereafter. Vitals signs will be charted at the PO clinical follow-up visit (between Day 14 and 30), and at termination of study (day 60±10). An ECG will be performed at baseline.
Vaccinia immunoglobulin (or equivalent) will be available for use in case of a severe toxicity related to GL-ONC1. (Please see Appendix 3 for guidelines for the use of VIG.)

Tumor evaluation:
Evaluation of tumor status for all patients will be conducted by chest CT scans at baseline (within 14 days of treatment) and at termination of study (day 60±10) based on RECIST criteria and modified RECIST (for mesothelioma). Optimally the initial scan will be done after drainage of the pleural space, unless for reasons of clinical safety, a CT scan is required to characterize the location and extent of the effusion prior to drainage.

Assessment of Viral Appearance in Tumor:
Unless medically contraindicated, patients will undergo Video-Assisted Thoracic Surgery (VATS) with pleural biopsies to assess for green fluorescent protein (GFP) viral expression in tumor and surrounding tissues, and if appropriate, to perform pleurodesis at 2-7 days after intrapleural instillation of virus. For patients who receive multiple infusions, a VATS with pleural biopsies with or without pleurodesis will be performed following either the 3rd infusion at days 2 – 9, unless post-infusion toxicity after doses 1 or 2 dictates with holding planned subsequent doses. Random pleural biopsies and GFP-directed biopsies will be performed to allow for assessment of viral presence. Viral plaque assays (VPA) will be performed in tumor biopsies. Immunohistochemical (IHC), staining for GL-ONC1 and beta-glucuronidase testing will be performed on both GFP (-) and (+) areas at videothoracoscopy (if applicable).

Archival tissue samples (pre and post treatment) may be collected (with IRB#: 06-107 consent *yes' to first 3 questions in #IRB 12-169 consent*) to be analayzed for additional assays (e.g. Ki67), which may identify an anti-tumor effect from GL-ONC1 treatment.
Viral detection after treatment:
Single Infusion (Cohorts +1 to +4)
Patients will undergo serial sampling of blood, sputum, urine samples and pleural drainage for evaluation of viral particles by VPA immediately before treatment, and on days 2 and 3 after treatment.

Multiple Infusions (Cohorts +5 to IIa):
Viral Plaque Assays
Patients will undergo serial sampling of blood, sputum, urine samples and pleural drainage for evaluation of viral particles by VPA at baseline (prior to treatment), and on Days 2 and 3, and daily for 2 days following the final treatment.

Beta-glucuronidase
Patients will undergo serial sampling of pleural drainage for evaluation of beta-glucuronidase at baseline (prior to treatment), and on Days 2 and 3, and daily for 2 days following the final treatment.
Neutralizing Antibodies:
A neutralizing antibody assay will be used to detect anti-vaccinia antibody titers at baseline (within 14 days prior to treatment), and at termination of study (at day 60 ±10).

Immunophenotyping and cytokine analysis (immune response):
Analysis of lymphocyte subsets (e.g., CD4, CD8, CD19, CD25, CD56, CD69) and cytokine profile (plasma-IFN gamma, TNF and IL-1) will be performed on blood at baseline (within 14 days prior to treatment) and on Days 2 and 4, and at Day 60 (± 10 days).

In case of study procedures occurring on weekends/holidays or other logistical issues, a leeway of +/- 3 days is considered to be included for all time points stated in this protocol, if not stated otherwise. If a medical procedure or test which is conducted before signed consent as standard of care for a patient, that would have occurred regardless of whether patient considered participation in this clinical trial, may be used in place of a screening procedure (e.g., CT scans). If a patient is unable to physically produce a sample (urine, blood, sputum, and/or pleural fluid) necessary for any of the viral detection assays pre or post treatment, samples will attempted to be obtained within a +/- 3 day window however it will not constitute a protocol violation.

For patients who experience post-infusion toxicities after doses 1 or 2 that dictate with holding planned subsequent doses, the schedule of specimen collections for viral detection assays (referenced above) will be modified. Specimens will likely be collected pretreatment (baseline) and up to two days after the final treatment of GL-ONC1.

Number of patients:
A precise sample size cannot be defined as it is dependent on the observed toxicity, as well as doses chosen for de-escalation if this is necessary. Cohorts of three to six patients will be treated at each dose level up to a maximum of 1×10^{10} pfu, or until the MTD has been reached.

With or without identifying the MTD, the recommended dose level will be expanded by 3 additional patients to a maximum of 6 total patients who will be treated to better define the tolerability, viral replication and pharmacodynamics of the dose level and schedule. If no de-escalation is necessary, the maximum number of evaluable patients will be 25 (12 patients already evaluated at D1-D4 at the time of this amendment, plus one patient in treatment in the originally planned dose expansion cohort at D4, plus a maximum of 3x3 = 9 patients at D5-D7, plus 3 additional patients in the expansion cohort). Considering possible de-escalation at intermediate doses, the maximum number of evaluable patients will be 34.

2.0 OBJECTIVES AND SCIENTIFIC AIMS

Primary Objective:

- To determine the recommended Phase II dose of a genetically attenuated vaccinia virus, GL-ONC1, when administered to patients with malignant pleural effusion (primary non-small-cell lung carcinoma, mesothelioma, and other histologies).

Secondary Objectives:

- To detect virus in body fluids;
- To document infection of tumor by virus through GFP-based endoscopy, viral plaque assays, immunohistochemical staining, and beta-glucuronidase assay;
- To describe the immune response generated to GL-ONC1;
- To determine the feasibility, safety and tolerability of the intrapleural administration
- To document possible therapeutic efficacy.

3.0 BACKGROUND AND RATIONALE

Currently, a number of oncolytic viruses are at various stages of clinical development. Concerning this worldwide effort, the ideal oncolytic virus would be tumor-specific in its action with negligible systemic or local toxicities, and would be able to infect or exert cytopathic effects on all tumor cells within an organism. It would be easily manufactured and stored for widespread use, and would be able to circumvent potential immune down regulation. Further, the need for genetic manipulation of the virus would be minimal or, ideally, it would be a wild type virus. None of the viruses currently under investigation has the ability to fulfill this idealistic viral phenotype. However, recent work has investigated the role of vaccinia virus as a potential oncolytic virus that may satisfy many of the above criteria.

3.1 Vaccinia viruses & GL-ONC1

Vaccinia virus (VACV) is the prototype member of the genus Orthopoxvirus (subfamily Chordopoxviridae; family Poxviridae). VACV has a large and complex particle containing a single linear double-stranded DNA genome of 186 kb with inverted terminal repeats and terminal hairpin loops. The DNA genome of a number of strains of VACV has been sequenced and encoding approximately 150-200 proteins. Many essential genes are found within the more highly conserved central portion of the genome, while genes that are non-essential for replication and morphogenesis (in cell culture) are located closer to the ends. Genes are transcribed from both DNA strands and gene expression occurs in two phases - early and late. VACV replicates entirely in the cytoplasm of infected cells and the virus particle contains the enzymes (RNA polymerase, polyA polymerase, capping enzyme and methylating enzymes) required for the synthesis of capped (methylated) polyadenylated mRNAs. This particular characteristic of VACV mitigates its potential for mutagenesis by incorporation into the host genome.

Vaccinia virus has many characteristics desirable in an oncolytic virus for clinical applications: 1) short, well-characterized life cycle, spreading very rapidly from cell-to-cell; 2) highly cytolytic for a broad range of tumor cell types; 3) a large insertion capacity (> 25 kb) for the expression of exogenous genes; 4) high genetic stability; 5) amenable to large scale production of high levels of infectious virus; 6) does not have a natural host and does not cause any known diseases in humans; 7) remains in the cytoplasm and does not enter the host cell nucleus during the entire life cycle, and thus does not integrate into the host genome; 8) used extensively as smallpox vaccine in millions of people with well documented side effects; 9) drugs (e.g., vaccinia immunoglobulin, cidofovir, ST-246, etc.) are available to effectively treat any potential vaccinia infections, and

10) safely administered intravenously in a previous Phase I clinical trial.

Genelux has genetically engineered a vaccinia virus, designated as GLV-1h68 (proprietary name GL-ONC1). GLV-1h68 was used in most of the efficacy experiments in animals. The GMP-derived material of this same virus is called GL-ONC1. GL-ONC1 has been used primarily for all safety pharmacology and toxicological experiments, performed at the Bioservice Scientific Laboratories (BSL), as well as in *in vitro* potency comparisons (in cell cultures) and *in vivo* potency comparisons (in tumorous animals). GLV-1h68 was derived from the LIVP strain by inserting *RUC-GFP* (a fusion gene of *Renilla* luciferase and green fluorescent protein), *LacZ* (beta-galactosidase), and *gusA* (beta-glucuronidase) expression cassettes into *F14.5L* (located between *F14L* and *F15L*), thymidine kinase (*TK*), and hemagglutinin (*HA*) loci, respectively. Disruption of these non-essential genes and expression of the foreign gene expression cassettes not only attenuated the virus but also enhanced its tumor-specific targeting.

In preclinical testing, the virus showed diagnostic and therapeutic effects. In particular, systemic delivery of this virus resulted in the complete eradication or significant growth inhibition of several types of large subcutaneous xenograft tumors (including breast, lung, pancreatic, ovarian, colon, and prostate) in nude mice with no significant adverse effects.

3.2 Non-clinical efficacy studies

In vitro, a large panel of tumor cells could be killed efficiently by GLV-1h68 (see examples in table below).

Cell line	Human tumor type	Percent of cells killed @ 72 hpi	
		MOI = 0.1	MOI = 1.0
PC-3	Prostate carcinoma	70%	95%
DU145	Prostate carcinoma	99%	100%
A549	Lung carcinoma	60%	-
GI-101A	Breast carcinoma	25%	-
8505C	Anaplastic thyroid carcinoma	-	60%
8305C	Anaplastic thyroid carcinoma	10%	60%
KAT4C	Anaplastic thyroid carcinoma	-	60%
KAT4	Anaplastic thyroid carcinoma	10%	75%
MSTO-211H	Malignant mesothelioma	65%	80%
SCC15	Squamous cell carcinoma	40%	80%
SCC25	Squamous cell carcinoma	10%	65%
MSKQLL1	Squamous cell carcinoma	30%	75%
MSKQLL2	Squamous cell carcinoma	30%	85%
CAPAN-2	Pancreatic carcinoma	15%	65%

In order to assess efficacy, Genelux and collaborating laboratories administered various doses of GLV-1h68 to mice and rats carrying several human tumor xenografts, including breast, lung, colon, prostate, ovarian and pancreatic tumors. Various types of murine tumor lines were also used. Because eradication of tumor tissue was considered a main parameter of efficacy, tumor regression was monitored using light emission and by measuring tumor volume using a digital caliper. Intravenous, intratumoral or intrapleural delivery of this virus resulted in the complete eradication or significant growth inhibition of several types of large subcutaneous xenograft tumors (including breast, lung, colon, prostate, ovarian, pancreatic, thyroid, and skin), and an orthotopic mesothelioma model in nude mice with no significant adverse effects.

3.2.1 Effect of GL-ONC1 on breast xenografts in mice

During the course of the study, GLV-1h68 caused a remarkable decrease in the size of human breast tumor subcutaneous xenografts in nude mice. More detailed examination showed that, after intravenous injection of the virus, breast tumors (500 mm^3 initially) showed a three-phase growth pattern. In phase I (growth phase), tumors increased in volume until they reached approximately 1,500-2,000 mm^3. A slight growth stimulation (or size enlargement) of tumors upon virus colonization was observed in this phase in comparison to control mice with no virus infection. Fourteen days after virus injection, the tumor growth was arrested (phase II, inhibitory phase) when compared to the uninfected control group. The inhibitory phase ended approximately four weeks after virus infection. Thereafter, virus-colonized tumors began to regress rapidly. The regression phase (phase III, regression phase) continued and resulted in complete tumor elimination in more than 95% of mice (n > 50) within 130 days after virus injection.

A nearly identical three-phase tumor regression pattern was observed when a single dose of 1×10^5, 1×10^6 or 1×10^7 pfu of GLV-1h68 was delivered intravenously, indicating that a 1×10^5 pfu dose was sufficient to achieve tumor colonization and tumor therapy efficacy in nude mice. In this model, multiple doses yielded similar tumor regression patterns. No release of infectious viral particles was found in the blood 14 days after virus injection or during subsequent tumor regression stages.

The GLV-1h68-mediated β-galactosidase expression in regressing tumors was also analyzed with immunohistochemical (IHC) staining. The IHC staining of β-galactosidase was visible in the tumor sections 28 days after virus infection. However, at the end of phase III, in parallel with extinction of GFP fluorescence, β-galactosidase staining from infected tumor sections was completely absent. Other than transiently in unhealed cutaneous wounds (such as ear-tag wounds, mice bite marks, etc.), neither GFP fluorescence nor β-galactosidase staining could be seen outside of tumor tissues.

3.2.2 Effect of GLV-1h68/GL-ONC1 on lung xenograft growth

The therapeutic effect of GL-ONC1 on the progression of lung tumor subcutaneous xenografts was evaluated *in vivo* by measuring tumor volumes at various time points. Tumors were established by subcutaneously injecting A549 human lung carcinoma cells on right lateral thighs of male nude mice. Thirty-six days after tumor cell implantation, one group of seven mice was injected intravenously with 1×10^6 pfu GLV-1h68. A second group of seven mice was injected intravenously with 2×10^7 pfu GLV-1h68, and a control group of four mice did not receive any treatment. Tumor inhibition was more pronounced with the higher dose of GLV-1h68 (60% growth inhibition) than with the lower dose (45% growth inhibition) at 48 days after virus treatment.

3.2.3 Effect of GLV-1h68 on thyroid xenograft growth

In this study, the efficacy of intratumorally injected GLV-1h68 was examined in two human Anaplastic thyroid carcinoma (ATC) xenograft models (8505C, DRO90-1). It was found that GLV-1h68 replicated efficiently and specifically within the ATC xenografts. A single intratumoral injection significantly inhibited ATC growth. Tumor volume progression was significantly impeded for mice treated with GLV-1h68 (5×10^6 pfu) as compared to control tumors (n = 5 per group) for both 8505C (p = 0.028 at day 45, t-test), and DRO90-1 (p = 0.03 at day 18, t-test) tumors.

3.2.4 Effect of GLV-1h68 on prostate xenograft growth

The therapeutic effect of GL-ONC1 on the progression of prostate tumors was evaluated in two tumor models

with male nude mice by measuring the volume of the tumor at various time points. In the PC-3 model, tumor growth was 90% slower in the virus-treated group (with a single, intravenous injection of GL-1h68 at 5×10^7 pfu per animal) than in the non-treated control group at 35 days after virus injection. In the DU145 model, tumor growth was 80% slower at 43 days after virus injection.

3.2.5 Effect of GLV-1h68 on ovarian tumor growth

The therapeutic effect of GL-ONC1 on the progression of human ovarian OVCAR-3 tumors was evaluated in a direct *in vivo* study with female nude mice by measuring the volume of the tumor at various time points. Administration of GLV-1h68 intravenously at 1×10^7 pfu per animal was able to slow tumor growth by 65% at 23 days after virus injection.

3.2.6 Effect of GLV-1h68 on pancreatic xenograft growth

The therapeutic effect of GL-ONC1 on the progression of human pancreatic tumors was evaluated in direct *in vivo* studies in male nude mice by measuring the volume of the tumor at various time points.

Two separate experiments were carried out:

In PANC-1 human pancreatic carcinoma xenograft model, a group of mice was injected intravenously with a single dose of 2×10^6 pfu of GLV-1h68, and a second group was treated with five repeated injections of 5 mg/kg cisplatin. The control group of mice was treated with PBS. Although cisplatin significantly slowed the growth rate of the pancreatic tumors compared to untreated controls, it was unable to arrest tumor growth. GLV-1h68, on the other hand, caused sustained shrinkage of pancreatic tumors as early as 29 days after virus injection. Complete tumor eradication was observed approximately two months after virus injection.

In MIA-PaCa2 human pancreatic carcinoma xenograft model, a group of mice was injected with 5×10^6 pfu GLV-1h68 in the femoral vein 31 days after tumor cell implantation, and the control group of mice was treated with PBS. GLV-1h68 caused marked inhibition of the MIA-PaCa2 pancreatic tumors (an 80% growth inhibition by day 25). The fact that tumor shrinkage was demonstrated in more than one type of pancreatic cancer indicates that the therapeutic effect of GLV-1h68 is not limited to a particular subgroup of pancreatic cancers.

3.2.7 Effect of GL-ONC1 on mesothelioma xenograft growth

Malignant pleural mesothelioma (MPM) is a fatal disease with a median survival of less than 14 months. In an orthotopic model, GLV-1h68, applied intrapleurally, effectively prevented development of cachexia and tumor-related morbidity, reduced tumor burden, and cured MPM in both early and late treatment groups. On day 10 after GLV-1h68 (1×10^7 pfu) administration, three of five late treatment, and two of five early treatment animals, were completely cured of their disease as seen on gross examination; while the other virally treated animals had minimal mediastinal disease.

3.2.8 Effect of GL-ONC1 on melanoma xenograft growth

The purpose of this study was to evaluate the safety and therapeutic efficacy of GLV-1h68 (5×10^6 pfu/ animal) following a single intravenous injection in male nude mice (n = 6-8) bearing subcutaneous (s.c.) 888-MEL human melanoma tumors. GLV-1h68 treatment resulted in a tumor growth inhibition of ~80% at the end of the first month after virus administration. Comparing to the mortality and high tumor growth rate in the untreated group, GLV-1h68 treatment seemed to help prolong the survival of mice with melanoma tumors.

3.3 Non-clinical safety studies

3.3.1 Biodistribution

Genelux laboratories and a contracted GLP laboratory, Bioservices Science Laboratories GmbH (BSL) tested the biodistribution of GLV-1h68 in both mouse and rat species. After single intravenous injections of varying doses of GLV-1h68/GL-ONC1, animals were monitored, then sacrificed at various time points to examine the distribution of the virus in organs, blood and feces. The experiments showed that the virus was cleared from the healthy tissues of most of the animals, while continuing to colonize in the tumors.

3.3.2 Persistence

Persistence of GL-ONC1 was measured using a medium-dose (1×10^7 pfu) of the virus in immunocompetent rats.

The aim of the test was to determine the devolution of the distribution of GL-ONC1. Defined organs were excised from three male and three female animals 1, 2, 3, 4, 5 and 6 months after viral application.

Samples were taken from the organs (kidney, lung, spleen, brain, ovaries/testes, liver, heart, and bladder); EDTA-blood and feces were studied using VPA and qPCR analyses. The VPA data measured in Genelux laboratories in Bernried, Germany, established complete absence of virus at each time point measured in all organs assessed.

3.3.3 Safety Pharmacology

3.3.3.1 Effects of GL-ONC1 on cardiovascular parameters in anaesthetized rats

GL-ONC1 was injected intravenously at a dose of 1×10^8 pfu per animal in a volume of 1.6 mL. There were no biologically relevant differences with respect to systolic or diastolic arterial blood pressures and heart rate measured between test and control animals, nor did the calculation of mean arterial blood pressure and heart rate reveal any relevant changes. The ECG did not reveal any arrhythmias.

3.3.3.2 Effects of GL-ONC1 on respiratory parameters in anaesthetized rats

GL-ONC1 was injected intravenously at a dose of 1×10^8 pfu per animal in a volume of 1.6 mL. No biologically relevant differences concerning respiratory parameters, with respect to respiratory rate and tidal volume were observed between test and control animals, nor did the calculation of the respiratory minute volume reveal any relevant changes. There were no significant changes in blood pressure measurements.

3.3.3.3 Effects of GL-ONC1 on the central nervous system in the rat

The study was performed in order to evaluate the influence of GL-ONC1 on central and autonomic nervous systems function, assessed according to the IRWIN screening test. Additionally, the psycho-motor behavior and spontaneous activity were assessed in the open field.

GL-ONC1 was injected intravenously at a dose of 1×10^8 pfu per animal in a volume of 1.6 mL. Neither test nor control animals showed abnormalities in functional and behavioral examination at any observation time. No abnormalities were recorded in gait, posture, palpebral closure, lacrimation, pileroction, arousal and vocalization. No convulsions, tremors, stereotypic or abnormal behaviors were observed. Responses to reflex testing were normal. Pupil response and ophthalmologic examination did not reveal any abnormalities.

3.4 Toxicity

Toxicology for GL-ONC1 was studied by intravenously injecting varying doses into mice and rats, both non-

tumorous and tumor-bearing. In each study, gross and histopathological examinations were carried out for all animals.

3.4.1 Single-Dose Toxicity in mice and rats

Acute toxicity in immunocompetent albino mice was tested by intravenously injecting GL-ONC1 into three groups of five male and five female mice. The low-dose group received 1×10^5 pfu per animal and the medium dose group received 1×10^7 pfu per animal. The highest dose, which was 1×10^8 pfu per animal, was administered through two consecutive injections over a period of five hours due to the large inoculum volume. Two days post-injection, one male animal from the 1×10^8 pfu dose was found dead. Two days post-injection, three female test animals from the high-dose group were found dead. No other compound-related mortalities, and no other signs of toxicity were recorded within 14 days post-injection for any dose or control groups.

Acute toxicity was tested by intravenously injecting GL-ONC1 into three groups of five male and five female immunocompetent Wistar rats. The low dose group received 1×10^5 pfu per animal and the medium dose group received 1×10^7 pfu per animal and the high dose group received 1×10^8 pfu per animal.

Animals were observed for 14 days after dosing. A careful clinical examination was made once a day. At the end of the observation period, the animals were sacrificed and necropsy was carried out to record gross pathological changes. All animals survived without showing any clinical symptoms.

3.4.2 Repeat-Dose Toxicity in rats

Macroscopic observations recorded at necropsy and histological slides were evaluated from main and recovery animals of a 28-day intravenous toxicity study in Wistar rats. Each animal received a twice-weekly administration of GL-ONC1 at dose levels of 0 (vehicle control), 1×10^5, 1×10^7 or 1×10^8 pfu, followed by a two-week treatment-free recovery period.

Treatment with GL-ONC1 caused histopathological findings in the liver, lung, axillary lymph node and spleen. In the liver, diffuse perilobular single cell necrosis and fibrosis, as well as a slightly higher degree of portal mononuclear cell infiltrate, with eosinophilic granulocytes, was seen in high dose male and female rats. In some animals, the change was associated with increased mitosis. Liver changes were considered to be toxicologically relevant. They were partially reversible after the two-week treatment-free period.

At the end of the recovery period, perilobular single cell necrosis and fibrosis were almost completely reversed in the high dose males and partially reversed in the high dose females. The same was true for portal infiltrates. The frequency of mitotic events was no longer increasing. Multifocal anisokaryosis in two out of five high dose females was considered to be indicative of hepatic reconstitution.

An increased incidence of perivascular mixed cell infiltrates in the lung was observed in the high dose rats as well as in the medium dose female rats and was considered to be toxicologically relevant. The change was partially reversed after the two-week recovery period.

In the axillary lymph node and spleen, germinal center development was increased in a dose-dependent manner in all treated groups, males and females. In the spleen, plasma cell aggregations were noted in males and females of all treatment groups, and an atrophy of the marginal zone was seen in the high dose groups. Changes of lymphoid organs were considered to reflect the heightened immune response to the test article administration and were partially reversed after the two-week recovery period.

3.4.3 Toxicology and bio-distribution in tumor-bearing mice

A GLP study was performed by Comparative Biosciences, Inc., to evaluate the toxicity of GL-ONC1 following a single intratumoral injection in tumor-bearing athymic nude mice and a single intraperitoneal injection in non-tumor-bearing athymic nude mice and non-tumor-bearing wild-type mice. GL-ONC1 was dosed at three different levels (1×10^6, 3×10^6, or 1×10^7 pfu/animal). Two mortalities (one male, one female) occurred only in the mid-dose (but not high-dose) tumor-bearing nude mice; these deaths were not considered to be test article-related. All changes in hematology and chemistry parameter were generally mild and within reference ranges except that the potassium increases in some mice could not determined (if beyond 10.0 mEq/L). There were no indications of adverse effects in test article-dosed mice as judged by clinical signs, body weights, food consumption or tumor growth. Some dose-dependent changes were observed in clinical pathology and organ weights. The majority of the clinical pathology changes was mild and within reference ranges. Histopathology did not reveal any dose-related effects at either seven day or two months sacrifice.

Therefore, there was no evidence of ill effects of the test article as judged by in-life or necropsy observations.

3.4.4 Horizontal transmissibility studies

To study the potential transmissibility of the virus, the prior two studies (conducted at BSL, Germany) each included an additional group of three male and three female animals that were caged together with the animals of the high dose group.

In both mouse and rat studies, the animals were observed for 21 days after dosing. A careful clinical examination was made once a day. At the end of the observation period, the animals were sacrificed and necropsy was carried out to record gross pathological changes.

In both studies, there were no sign of horizontal transmissibility. All animals survived. Normal weight gain was observed and no detectable amounts of the virus were found in the organs, blood or feces.

3.5 Clinical Experience

3.5.1 Clinical case reports

A study in the February 1987 issue of the *Japanese Journal of Experimental Medicine* reported successful treatment of a 67-year-old male multiple myeloma patient using an intravenous injection of the AS strain of vaccinia virus. By the 106th day of treatment, the NK cell activity had risen to 33% from the initial 20% on the day 10. No adverse effects were observed. The patient remained in remission three years after treatment.[1] Later that year, the same research group reported that they had achieved favorable anti-tumor effects using the AS strain on two cases of advanced adenocarcinoma without observing any adverse reactions.[2]

An earlier 1978 study reported that a 78-year-old man with untreated chronic lymphocytic leukemia went into complete remission after revaccination for smallpox. He endured a severe local reaction and generalized rash following the vaccination, which responded well to treatment with human vaccinia immune globulin.[3] Other medical practitioners have confirmed these findings with responses of patients vaccinated for smallpox.[4]

Historically, wild-type vaccinia virus has also been studied, whether deliberately or not, for potential treatment of human cancers. A wild-type Wyeth strain was used recently in a clinical trial for recurrent melanoma. For more information on these trials, see references.

Several Phase I studies have used recombinant vaccinia virus for oncolytic virotherapy. In general, the agents have been well-tolerated and effective at inducing CEA-specific cytotoxic T cell responses, a common goal in

VACV studies.

3.5.2 Smallpox vaccine

As the smallpox vaccine, vaccinia virus itself has a long history of clinical use from which the medical and research community can draw safety data. In 1968, when the vaccine was still a common immunization tool, epidemiologists collected data on adverse events related to vaccination in both a national and a ten-state survey.[5,6] The results of their work are presented in the following table:

Smallpox Vaccine: Adverse Event Rates, 1968
(number per million vaccines)

	NATIONAL SURVEY		TEN-STATE SURVEY	
	**All primary (i.e., first-time) vaccines**	**Vaccines ≥ 1 yr old**	**All primary (i.e., first-time) vaccines**	**Vaccines ≥ 1 yr old**
	**Serious, but not life-threatening reactions:**			
Inadvertent Inoculation	25.4	27.1	529.2	532.0
Generalized Vaccinia	23.4	17.7	241.5	222.8
Erythema Multiforme	N/A	N/A	164.6	131.3
**Total number of serious, but not life-threatening reactions:**	**48.8**		**935.3***	
	**Life-threatening reactions:**			
Postvaccinal Encephalitis	2.9	2.4	12.3	8.6
Progressive Vaccinia (Vaccinia Necrosum)	0.9	1.0	1.5	1.7
Eczema Vaccinatum	10.4	10.6	38.5	41.5
**Total number of life-threatening reactions:**	**14.2***		**52.3***	
**Deaths:**	**1.1***	0.6	1.5	None Reported

***Adverse event statistics cited in document are marked with an asterisk.

3.5.3 Vaccinia virus for cancer therapy

Lattime and Mastrangelo have done significant amount of ground work using vaccinia virus in cancer gene therapy in human patients.[7,8,9,10] In some of these studies, injection of vaccinia recombinants expressing immune-stimulating cytokines, such as GM-CSF, into patients were shown to be well tolerated. More recently, additional safety data have been obtained in clinical studies using the same vaccinia-GM-CSF.[11,12]

3.5.4 Phase I study on the intravenous administration of GL-ONC1

GL-ONC1 has been investigated in a first-in-human Phase I trial performed in the United Kingdom in patients diagnosed with advanced solid tumors as a monotherapy delivered intravenously or as bolus infusion.

Protocol Number	Indication	Administration Plan	Dose	Number of Patients Treated
GL-ONC1-002/MA	Advanced solid tumors	Monotherapy as intravenous or bolus infusion	Treatment cycles ranged from 28 days, 2 weeks	43

(United Kingdom		GL-ONC1 administered either as single dose/cycle, or up to 3 consecutive doses/cycle.	and 1 week<br>1×10^5 pfu to 5×10^9 pfu (starting dose based on pre-clinical based results)	

Safety: The data presented below represents adverse reactions to GL-ONC1 reported to date (N=number of reported adverse reactions per reaction) for all reactions with ≥ 5 occurrences.

**Reaction**	**Number of Occurrences N = 249 (N%)**
Pyrexia	48 (19.3%)
Chills	23 (9.2%)
Lymphopenia	20 (8.0%)
Rigors	18 (7.4%)
Hypotension	16 (6.4%)
Fatigue	14 (5.6%)
Vomiting	13 (5.2%)
Nausea	12 (4.8%)
APTT increased	11 (4.4%)
Tachycardia	11 (4.4%)
GGT increased	11 (4.4%)
Blood alkaline phosphatase increased	10 (4.0%)
AST increased	9 (3.6%)
ALT increased	8 (3.2%)
C-reactive protein increased	8 (3.2%)
Low oxygen saturation	7 (2.8%)
Myalgia	7 (2.8%)
Rash	6 (2.4%)
Creatinine increased	5 (2.0%)
Thrombocytopenia	5 (2.0%)

Serious Adverse Events (SAEs), Results of Viral Shedding, PK and PD Laboratory Testing and Response to Treatment

GL-ONC1-002/MA (United Kingdom: Phase I Advanced solid tumors; NCT00794131):

Phase I: Sixteen SAEs were reported for 9 out of 27 treated patients.

Five SAEs were determined by the Investigator to have a degree of relatedness to GL-ONC1:

- *Cohort 2 Patient (single dose per cycle of 1 × 10^6 pfu):* Mild (Grade 1) pyrexia which resolved within 3 days which occurred while this patient was hospitalized for treatment of progressive disease unrelated to GL-ONC1 which led to the death of patient (unrelated to treatment with GL-ONC1).
- *Cohort 5 (single dose per cycle of 1 × 10^9 pfu):* One patient experienced severe (Grade 3) leg stiffness and pain, and was also treated for a severe arterial embolism in the left leg and was withdrawn after Cycle 6 from receiving further treatment with ongoing symptoms at the time of withdrawal.
- *Cohort 5a (single dose per cycle of 1 × 10^9 pfu):* Moderate (Grade 2) thrombocytopenia was reported for a patient in this cohort which resolved in one day without sequelae and did not require concomitant medication.

Eleven SAEs determined not related to GL-ONC1 treatment included:

- *Cohort 2 Patient (single dose per cycle of 1 × 10^6 pfu):* Two cases of moderate to severe infected metatastic skin lesions were reported for one patient.
- *Cohort 2 Patient (single dose per cycle of 1 × 10^6 pfu):* A patient was hospitalized for treatment of life threatening (Grade 4) disease progression from which the patient died which was determined to be unrelated to treatment with GL-ONC1.
- *Cohort 3 (single dose per cycle of 1 × 10^6 pfu)*: This patient was determined to have brain metastatsis following intiation of study drug treatment and the patient was withdrawn from treatment after one cycle of GL-ONC1 and later died from progressive disease unrelated to GL-ONC1 treatment.
- *Cohort 3 (single dose per cycle of 1 × 10^6 pfu)*: A patient was hospitalized for treatment of mild anorexia.
- *Cohort 5a (single dose per cycle of 1 × 10^9 pfu):* A patient experienced severe bronchopnumonia, severe increase in neutrophils and white blood cells, and severe hypotension which ultimately lead to the death unrelated to treatment with GL-ONC1.
- Cohort 5b *(single dose per cycle of 3 × 10^9 pfu):* This patient was hospitalized for treatment of tumor bleeding and subsequently died after being withdrawn from treatment following receiving 1 cycle of GL-ONC1. Death was unrelated to treatment with GL-ONC1.
- *Cohort 6 (three consecutive doses per cycle of 1.667 × 10^7 pfu):* Patient was hospitalized for treatment of severe pneumonia with outcome of death not related to treatment with GL-ONC1.

Phase Ib: Nine SAEs were reported for 6 out of 16 treated patients.

One SAE was determined by the Investigator to have a degree of relatedness to GL-ONC1:

- *Cohort 8c (Cycle 1 at three consecutive doses per cycle of 1.667 × 10^9 pfu; Cycles 2-6 single doses at 5 × 10^9 pfu):* A patient diagnosed with esophogeal carcinoma was hospitalized after developing an treacheal airway obstruction following the symptomatic growth of disease during the later cycles of GL-ONC1 treatment, and a tracheal stent was put in place. The Investigator determined that this development was severe and possibly related to treatment with GL-ONC1 due to immunotherapy/virotherpay causing transient tumor swelling before regression. The Medical Monitor's opinion was that this event was due to the typical progression of the disase and felt that the event was unlikely related. Due to the tracheal obstruction not listed as a known toxicity for GL-ONC1, this event was reported to U.S. and European regulatory authorities and clinical investigators as a Suspected Unexpected Serious Adverse Reaction (SUSAR).

Eight SAEs were determined not related to GL-ONC1 treatment which included:

- *Cohort Ib (single dose per cycle of 3 × 10^9 pfu):* Patient was hospitalized after developing severe cellulitis in the right leg unrelated to GL-ONC1 treatment with resolved without sequelae.
- *Cohort Ib (single dose per cycle of 3 × 10^9 pfu):* A patient was hospitalized for treatment of moderate nausea and vomiting and increased output from colostomy with did not require concomitant medication and resolved with sequelae.
- *Cohort 8 (three consecutive doses per cycle of 1.667 × 10^9 pfu):* Patient diagnosed with biliary duct cholangiocarcinoma was hospitalized for a severe rise in ALT with a mild increase in bilirubin which resolved without sequealae. Symptoms were determined to be unrelated to treatment with GL-ONC1.
- *Cohort 8 (three consecutive doses per cycle of 1.667 × 10^9 pfu):* This patient developed life-threatening ascending cholantitis which which was treated and resolved without sequelae.
- *Cohort 8a (Cycle 1 at three consecutive doses per cycle of 1.667 × 10^9 pfu; Cycles 2-6 single doses at 3 × 10^9 pfu):* It was discovered following initiation of treatment with GL-ONC1 that this patient had a localized bowel perforation of the left iliac fossa which as stable not requiring additioinal treatment other than observation. Patient was removed after receiving one GL-ONC1 treatment due to this discovery.
- *Cohort 8a (Cycle 1 at three consecutive doses per cycle of 1.667 × 10^9 pfu; Cycles 2-6 single doses at 3 × 10^9 pfu):* This patient was hospitalized for treatment of severe right-sided pleuritic chest pain, pulmonary embolism, infection of PICC-line, lower respiratory tract infection.

Viral Shedding:

Minimal viral shedding analyzed by Viral Plaque Assay (VPA) of blood, urine, stool and sputum was evident in patients treated in the higher doses (i.e., 1 × 10^9 pfu and to 5 × 10^9 pfu) for Cycle 1.

Vaccinia Virus Positive Skin Rash:

A maculopapular skin rash comprising of vaccinia pustules appeared in two patients during cycle 1 for Cohort 5 (1 × 10^9 pfu × 1 dose/cycle) and resolved without treatment at the end of Cycle 1. Some lesions were positive for GL-ONC1 viral plaque assay (VPA) and GFP imaging.

Virus Infected Tumor Tissue:

Immunohistochemical staining of tumor tissue exhibited clear evidence of viral infection in tumor tissue on Cycle 4 / Day 15 for 1 patient with head and neck cancer. Other results regarding VPA and qPCR analysis have tested tumor biopsy samples for 16 patients, albeit with negative results.

Pharmacokinetic and Pharmacodynamic Data:

Viral kinetics showed a dose-dependent increase of viral presence in the blood for the first 10 hours post treatment of Cycle 1 and 2 which was transient with viral DNA shown to quickly clear from blood circulation with the exception of one patient who had delayed clearance. It was also evident in Cohorts 3 to 5b, 6, 7, 8a and 8b that substantial levels of viral titer could be maintained transiently after the second cycle of treatment with the durations of transient viral presence in the blood similar to that seen after Cycle 1 and 2 treatments. Vaccinia virus neutralizing antibodies showed an increase in all but one patient. However, except for Cohort 1, the antibody titers plateaued and did not result in further increase after the second cycle of treatment. Comparison of neutralizing antibody (NAb) levels between patients with stable disease versus progressive disease showed that patient with objective evidence of stable disease had lower levels of NAb than patients with progressive disease. Conversely, patients with higher levels of beta-glucuronidase had stable disease versus lower levels in patients with progressive disease (p-0.14). Circulating tumor cells (CTC) were analysed in the Phase Ib portion of this trial which showed evidence of virus infected CTCs with GFP+ CTC cells in 5

treated patients.

Tumor Response to Treatment:

Thirteen patients from early cohorts to later dose cohorts had radiographic evidence by CT scans to have stable disease from 8, 12, 13, 24 weeks and up to 48 weeks from baseline tumor imaging.

SUMMARY

Overall, there were no deaths reported to have any causality to treatment with GL-ONC1 which was well tolerated. Early evidence of efficacy was also shown as well as the ability of the vaccinia virus to infect tumor tissue.

3.6 Rationale for development of GL-ONC1 as an anti-cancer agent

Pre-clinical studies on GL-ONC1 have demonstrated a high degree of efficacy in many tumor models and adequate safety in two animal species.

Viral replication occurred with high selectively in malignant tissues, resulting in effective tumor reduction/elimination, and improved survival of the test animals. Efficacy was even superior to cytotoxic drugs in several *in vivo* models.

No normal tissue damage was observed at doses associated with anti-tumor activity. These promising results warrant careful evaluation of safety in patients and assessment of signs of efficacy.

Based on extensive preclinical safety studies (for details, see Investigator's Brochure), exposure of rats and mice with intravenous doses of 1×10^8 and 1×10^7 pfu, respectively, were shown to be safe. Hence, the proposed starting dose of 1×10^7 pfu in humans is considered to have a strong likelihood of being well tolerated in patients.

Based upon the state-of-the-art research in cancer diagnosis/therapy, and the supplier's own experimental data, Genelux has assessed the following possibilities:

- High probability for demonstrating safety of human subjects when the product is administered according to the proposed clinical trial;
- No scientific evidence suggests that GL-ONC1 will revert to more virulent strains;
- No scientific evidence suggests that GL-ONC1 will lead to mutations in host cells, hence low risk;
- No scientific evidence suggests that systemic complications or diseases will occur due to administration of GL-ONC1, hence low risk;
- High probability that GL-ONC1 will specifically colonize tumors in subjects;
- Up to now, virus shedding into the environment has to be considered to occur with a remote likelihood; accordingly, there only is a low risk of transmission of infection to other individuals.
- High probability that the reporter genes will aid in *in vivo* imaging of tumors and metastases and *in vitro* detection of malignancies in biopsy specimens.
-

3.7. Rationale for the use of GL-ONC1 in malignant pleural mesothelioma and in other diseases with malignant pleural effusions

Malignant pleural mesothelioma (MPM) is a deadly disease with a great need for novel therapeutic options.

There are only approximately 3,000 MPM cases per year in the United States but worldwide MPM is a significant public health problem especially in developing countries where asbestos mining and use are unregulated. The median survival in untreated patients is 12 months or less. Because of the latency period between asbestos exposure and the development of MPM (>20 years), most patients are older and have medical co-morbidities that limit treatment options. Only 10-20% of patients are candidates for surgical resection and multimodality therapy. Patients who have early stage disease, good performance and cardiopulmonary function are currently offered induction platinum-based chemotherapy, followed by surgical resection (pleurectomy/decortication or extrapleural pneumonectomy) and adjuvant hemi- thoracic radiation (a treatment approach developed at MSKCC and now used internationally). However, even this intensive 6 month treatment strategy yields at best an approximate 20 percent survival in stages I and II disease and a median survival of about 16 months in stage III disease. For patients with more locally advanced or with metastatic disease or whose performance status and cardiopulmonary reserve preclude multimodality treatment, few treatment options exist, all palliative. Current standard frontline chemotherapy (cisplatin or carboplatin and pemetrexed) has only about a 30% response rate.

However, a plateau has been reached with use of standard therapies for MPM. Completely novel approaches are needed for in this disease for which overall survival remains very poor. During the past 20 years, clinical research in MPM has focused on optimizing standard therapies. Refinements in staging, patient selection, and surgical care and technique have substantially decreased operative mortality. Improvements in radiotherapy have increase the role of radiation in this disease and have made postoperative adjuvant radiotherapy standard of care. Clinical trials testing many chemotherapy regimens (e.g. cisplatin or carboplatin plus pemetrexed or gemcitabine, vinorelbine)

Since the 1970's MSKCC has been internationally recognized as center of excellence in clinical and translational research in MPH and is one of the major referral centers worldwide for clinical care of MPH patients. Drs. Rusch (Thoracic Surgery), Krug (Thoracic Oncology) Rosenzweig and Rimner (Radiation Oncology) have led major national and international trials in MPM and Dr. Ladanyi (Pathology) is recognized for major discoveries in the molecular features of this disease, including most recently the presence of BAP-1 mutations in approximately 25% of MPM. Drs. Adusumilli (Thoracic Surgery) and Sadelain (SKI, Center for Cell-Based Therapies) are currently developing an immunotherapy-based treatment program for MPM. Thus, testing completely novel approaches in the treatment of this difficult disease is an important part of the mission of the Thoracic DMT. Preclinical studies suggest that intrapleural GL-ONC1 is a promising approach to the treatment of MPM.

In preclinical studies,[13] GL-ONC1 is able to productively infect, replicate in, and lyse a variety of human malignant mesothelioma cell lines, including epithelioid, sarcomatoid, and biphasic subtypes. In animal studies, GL-ONC1 successfully localized, infected, and displayed detectable transgene expression in tumor tissues after intrapleural administration. Animals in both early and late treatment groups maintained normal weight and food intake whereas control animals developed marked cachexia over a period of 3 weeks after tumor inoculation. GL-ONC1 demonstrated effective cure rates and marked reduction of tumor burden in settings of microscopic and macroscopic pleural disease. In addition, intrapleural therapy with GL-ONC1 resulted in increased survival in both microscopic and macroscopic disease settings.

Importantly, although GL-ONC1 displayed successful treatment of pleural disease, it did not result in any signs of toxicity or significant infection of normal host tissues over a varied time period, from 24 hrs to 4 weeks after administration of virus. GLV-1h68 therapy was well tolerated by athymic mice and resulted in essentially no signs of toxicity and displayed no replicative ability in normal tissues. We are confident that GLV-1h68 would be non-toxic to humans as vaccinia virus of the same strain has been safely administered to millions of people

via the smallpox vaccine(see slide presentation by Dr. Rusch to Recombinant DNA Advisory Committee (RAC), June 2011).

The safety, feasibility and tolerability of GL-ONC1 has been further shown in a Phase I trial of GL-ONC1 administered intravenously to patients with advanced solid tumors the Royal Marsden Hospital, U.K. (see poster and slides of presentation to the RAC in June 2011 by Dr. Kevin Harrington, protocol PI). The most frequently reported adverse events were grades 1 and 2 fatigue and fever. In 23 patients, only 2 grade 3 events and no grade 4 or 5 events were reported. Repeated administrations of up to 8 cycles of GL-ONC1 were safe and tolerable with no evidence of autoimmunity. In this heavily pretreated patient population the best response was stable disease (by RECIST) in 7 patients. Based on this experience a Phase I trial of GL-ONC1 and radiation in patients with advanced head and neck cancers is currently underway at UCSD.

For the purposes of this Phase I trial at MSKCC we propose administrating GL-ONC1 intrapleurally to patients with MPM and but also to patients with malignant pleural effusions for whom no better standard therapies exist. Such patients usually have symptoms predominantly related to their pleural effusion requiring some form of intrapleural treatment (e.g. Pleurx catheter or pleurodesis) and similar poor long-term outcomes. Although most of the patients will be indentified through the Thoracic Oncology Service, two other medical oncologists (Dr. Jackie Bromberg of the Breast Service and Dr. Nancy Kemeny of the GI Service) who have previously collaborated with Dr. Yuman Fong (HPB Surgery Service) in protocols of this type will also be screening patients for inclusion in this trial and will participate as co-investigators. Selective participation by specific medical and surgical oncologists in this initial Phase I trial is considered advisable for reasons of patient safety and data quality.

3.8. Rationale for consecutive day infusions of GL-ONC1

The first patient was treated and consented to IRB# 12-169 on 2/1/2013. As of 4/15/2014, 14 patients have been consented and 13 patients have been treated on protocol. One patient was ultimately found to be ineligible as a malignant pleural tissue diagnosis could not be definitely established. The 14th accrual completed the fourth and final cohort ($3x10^9$ pfus of GL-ONC1) and this patient was treated on 4/15/2013. At this time, 12 patients are evaluable for the protocol. One patient will be treated in the previously written (IRB# 12-169 A(6)) expansion cohort of the protocol at dose level D4 ($3x10^9$). Following treatment of this patient will increasing the total number of accrued patients to IRB#12-169 to 15 and treated patients to 14. The treatment date for this patient is scheduled for 8/26/2014. At this time the patient's evaluable status is unable to be determined.

Definitely and probably treatment related toxicities have included (see chart below):

Toxicity_Definite & Probable	G1	G2	G3	G4	# of times	# of patients
Alanine aminotransferase increased		x	x		7	2
Alkaline phosphatase increased	x	x			6	2
Aspartate aminotransferase increased	x		x		7	2
Blood bilirubin increased	x				1	1
Chills	x				7	4
Fatigue	x				1	1
Fever	x	x			12	6
Flu like symptoms	x				4	4
Headache	x				1	1
Nausea	x				1	1
Pain	x				1	1
Sinus tachycardia		x			1	1
Sweating	x				2	2

With the completion of the treatment phase of patient 014, no DLTs have been experienced and no MTD has been established. In addition, no related and unexpected SAEs have occurred, thus demonstrating the feasibility and safety of single dose administration of GL-ONC1.

Additional evidence provides rational for dose escalation to consecutive daily doses of GL-ONC1.

Justification for dosing schedule of 3 consecutive daily doses:

Preclinical studies in animal models demonstrated that infection of tumor tissue and subsequent oncolysis by vaccinia virus treatment are dose dependent. Multiple daily doses of virus is feasible and well tolerated in animal models. It has been shown that three consecutive daily doses of virus is more effective at colonizing tumors than a single injection at the same dose level per injection. Infection of tumor tissue causes local inflammation, and oncolysis results in tumor antigen release, both of which lead to further immune activation. We anticipate that the higher the dose, the more oncolysis and immune activation, and the better the therapeutic outcome (Lin et al. 2008 & data not yet published).

In a phase I study of GL-ONC1 administered intravenously to patients with advanced solid organ cancers and in another phase I study of GL-ONC1 administered intraperitoneally to patients with peritoneal carcinomatosis, anti-vaccinia neutralizing antibody has been seen to develop quickly within the first 8-10 days, and to plateau thereafter in the blood and peritoneal fluid, respectively (data not yet published). Hence administering virus within the first week would help to bypass subsequent peak of anti-vaccinia immune response. We have also found in the intravenous route (i.e., systemic) clinical trial that 3-consecutive-day treatments with GL-ONC1 are well tolerated. Therefore, we anticipate consecutive days of virus treatment administered intrapleurally will also be feasible and well tolerated in patients. The highest single dose of vaccinia given I.V. has been 5x10^{9} pfu, and up to 4x10^{10} pfu cumulatively in an individual patient. No MTD has been reached in any of the clinical trials of GL-ONC1, either delivered systemically or regionally. In Cohort 5 as a starting point of the proposed protocol amendment , the daily dose will be at 3x10^{9} pfu and cumulatively for 3 days of 9x10^{9} pfu total, both of which are considerably lower than what has been tested in the i.v. route study (data not yet published).

In summary, our initial experience in protocol IRB# 12-169 demonstrates safety and tolerability of single dose administration of GL-ONC1. Additional studies, in vitro, in animals, and in patients suggests that consecutive daily doses may be more effective and apear to be safe. Hence we plan to dose escalate with consecutive daily doses as noted below:

Cohort	Dose **	Number of doses	Total volume of each injection
+5	3 x 10^{9}	3	Final volume of preparation will be 500 mL, to be administered as a bolus.
+6	6 x 10^{9}	3	
+7	1 x 10^{10}	3	
Phase IIa (expansion)	(selected dose)***	3	

3.9 Rationale for washing step pre GL-ONC1 infusion

In addition, the study sponsor (Genelux Corporation for GL-ONC1-002/MA United Kingdom Phase I clinical trial) has recommended that prior to the GL-ONC1 infusions, a washing step be performed via the pleural catheter after drainage of the pleural space and before instillation of GL-ONC1.

The rationale for the washing step is that MPE consists of tumor cells, immune cells and other innate immunity. One of the innate immunities is the complement system, which is an innate response for inactivation of virus, such as GL-ONC1. The infectivity of the virus is reduced by this binding either directly as a function of the binding itself or through subsequent interactions that the bound complement components may direct.

Therefore this could significantly reduce the available virus for infecting tumor cells. In an ex vivo testing of MPE samples from a patient, it has been shown that the complement component of MPE could significantly inhibit vaccinia virus activity. Another component that could inactivate vaccinia virus is anti-vaccinia antibody from past vaccination against smallpox. MPE also consists resident NK cells, which are known to be able to inhibit and clear vaccinia virus as an infection (Burshtyn et al., NK cells and poxvirus infection. Front Immunol. 2013;4:7). To enhance the availability and duration of virus presence in the MPE for infection of tumors, strategies to eliminate such inhibitory factors are important. One way is to include a washing step prior to GL-ONC1 treatment to physically remove such limiting factors and help to maximize the effectiveness of oncolytic viral therapy.

3.10 Additional tissue/fluid-based assays .

Archival tissue samples (pre and post treatment) may be collected (with IRB# 06-107 consent) to be analyzed for additional anti-tumor effects (e.g. Ki67). Comparing the post-treatment tumor biopsy to the pretreatment tumor biopsy would help to make a determination of a potential indication of anti-tumor effect from GL-ONC1 treatment.

Additionally, the sponsor (Genelux Coporation) has recommended that pleural biopsies & pleural fluid be tested for beta glucuronidase to establish additional potential evidence for virus colonization and virus replication in the tumor.

4.0 OVERVIEW OF STUDY DESIGN/INTERVENTION

4.1 Design

This is an open-label, dose-escalating, non-randomized, single-center Phase I therapeutic study of GL-ONC1 originally administered intrapleurally as a single dose and now escalating to three consecutive daily doses in patients with a diagnosis (histologically or cytologically documented) of malignant pleural effusions. The total number of patients studied will depend on the number of dose levels tested up to a maximum dose of 1×10^{10} pfu/mL, or until the maximum tolerated dose (MTD) is reached. If no de-escalation is necessary, the maximum number of evaluable patients will be 25 (12 patients already evaluated at D1-D4 at the time of this amendment, plus one patient in treatment in the originally planned dose expansion cohort at D4, plus a maximum of 3x3 = 9 patients at D5-D7, plus 3 additional patients in the expansion cohort). Considering possible de-escalation at intermediate doses, the maximum number of evaluable patients will be 34.

4.2 Intervention

The treatment schema for this protocol with study intervention can be found below and is included in Appendix1.

Study Calendar Laboratory Tests or Procedures	Screening		Day 1 (Repeat for Days 2 & 3)**							Day 2*	Day 3*	Day 4*	Day 5*	Days 14-30	Day 60(±10)
	Within 8 weeks	Within 14 days	Immediately before treatment	Treatment with protocol intervention	30(±15) minutes	60(±15) minutes	120(±15) minutes	8(±1) hours	16(±1) hours	Repeat Day 1**	Repeat Day 1**	Operating Room		PO Clinical Follow-up	End of Treatment Phase
Informed consent		•													
Medical history/Clinical Status & Physical Exam		•												•	•
Concomitant medication		•	•											•	•
12-lead ECG		•													
Vital signs (BP, HR, Temp)		•	•		•	•	•	•	•	•	•	•	•	•	•
ECOG/Zubrod Score			•							•	•	•	•		•
Brain Scan (MRI/CT) *st. IV non meso patients only	•														
Tumor Imaging (Chest CT)		•													•
Routine bloodwork		•								•	•	•	•	•	•
Serum Pregnancy test (is applicable)		•													
**Specimen collection for research:**															
• Pleural drainage			•							•	•	•	•		
• Bloodwork			•							•	•	•	•		•
• Urine & Sputum			•							•	•	•	•		
• Pleural biopsies*										•	•	•	•		
Video-Assisted Thoracic Surgery (VATS)*										•	•	•	•		
Toxicity Assessment			•		•	•	•	•	•	•	•	•	•	•	•

*VATS with pleural biopsies to be done between Days 2 and 9. See also Intervention section of protocol.
** For multi-dose cohorts, repeat Day 1 for Days 2 & 3 GL-ONC 1 infusion and post infusion timepoints

The primary objective of this study is to provide a dosing recommendation for subsequent Phase II studies. Three patients will be enrolled in each cohort at the dose levels shown in the table below in order to determine the Maximum Tolerated Dose (MTD). At the beginning of a new dose level, only one patient will be treated. The first patient in each cohort must be treated and complete 14 days of post-treatment evaluation prior to the treatment of the remaining two patients in that cohort. All patients treated in a cohort must be evaluated for 14 days prior to treatment of the next dose cohort. However, the next patients can be pre-screened and enrolled to be ready for the next treatment. Following DSMC review and approval, the first patient in the next dose cohort may receive his/her first cycle 14 days after the third patient in the previous dose cohort received his/her first cycle. Any patient entered on study and treated with GL-ONC1 must be observed for adverse events for a minimum of 14 days in ordered to be considered evaluable.

Table: Dose level by cohort (flat dose independent of body weight)

Cohort	Dose **	No. of doses	Total volume of each injection
-1*	1×10^6	1	Final volume of preparation will be 500 mL, to be administered as a bolus.
+1	1×10^7	1	
+2	1×10^8	1	
+3	1×10^9	1	
+4	3×10^9	1	
+5***	$3 x 10^9$	3	
+6***	$6 x 10^9$	3	
+7***	$1 x 10^{10}$	3	
Phase IIa*** (expansion)	(selected dose)***	3	

* Necessary only if a DLT is encountered at the initial dose level.
** Dose de-escalation, if needed, will be done to intermediate levels that are one-half log lower, unless DSMC advises for full dose de-escalation
***Cohorts 5 or 6 or 7 may be selected as the expansion cohort based on safety. Three additional patients will be treated at the selected dose for expansion.

4.3 Dose Limiting Toxicity (DLT)

Any of the following may qualify as a DLT if it is determined by MSKCC DSMC to be possibly, probably or definitely related to GL-ONC1, irrespective of whether the toxicity resolves (see CTCAE Version 4.0):

- ANC (absolute neutrophil count) < 1000 mm3 with a single temperature of > 38.3 degree Celsius (101 degree F) or a substained temperature of => 38 degree Celsius (100.4 F) for more than 1 hour (febrile neutropenia).
- Platelet count < 25 $x10^9$/L.
- Grade 3/4 neurotoxicity or cardiotoxicity.
- Any other drug-related, non-haematological Grade 3 toxicity lasting > 5 days or Grade 4 toxicity (including flu-like symptoms, nausea and vomiting, which take place despite appropriate prophylactic measures).

Any AE that leads to a discontinuation of GL-ONC1 infusion will be considered to be a potential DLT. If the MSKCC DSMC believes that any AE may qualify as a DLT, this determination will be documented in CRDB.

4.4 Maximum Tolerated Dose (MTD)

If 1 patient out of 3 in a dose group experiences a Dose Limiting Toxicity (DLT), 3 more patients will be added to that dose group. If 2 or more patients out of a dose group of 6 patients experience the any severe adverse reaction (grade 3 or 4 toxicity)with a determination of relatedness to GL-ONC1 (i.e., possibly, probably, definitely), it is considered that a DLT for that cohort has been reached. Dose de-escalation will be to an intermediate level of one-half log lower, unless DSMC advises for de-escalation to the next lower dose. If tolerated, that dose cohort will be the MTD. For cohorts 4 and 5, de-escalation to cohort 3 is a one-half log reduction. If de-escalation is required for either Cohort 6 or Cohort 7, dose reduction will be to the prior cohort dose, or to a dose level in between

With or without identifying the MTD determination, 3 additional patients may be added, such that a total of 6 patients may be treated at the MTD or the recommended Phase II dose to further assess the toxicity and tolerability of the prospective recommended dose level, to evaluate the biological effects of systemically administered GL-ONC1, and to study virus delivery. Any additional toxicities seen in these 3 patients will be discussed with DSMC and may lead to reevaluation of the dose recommended for future studies.

To meet the minimum safety requirement for the determination of MTD, patients must have completed treatment or have experienced a DLT. Patients who do not meet these criteria need to be replaced.

5.0 THERAPEUTIC/DIAGNOSTIC AGENTS

5.1 Manufacturing

**INN name**	Not assigned
**Internal product name**	GL-ONC1
**Substance**	Live recombinant vaccinia virus
**Description**	Concentrate solution to be diluted with Ringer's Lactate solution
**Route of administration**	Intrapleural
**Manufacturer**	IDT Biologika GmbH
**Development**	Genelux Corporation, San Diego, CA USA and Genelux GmbH, Bernried, Germany

Supplier of GL-ONC1

Genelux GmbH
Am Neuland 1
D-82347 Bernried, Germany
Tel: +49 8158 9223-21
Fax: +49 8158 9223-35

Genelux Corporation
3030 Bunker Hill Street, Suite 310
San Diego, CA 92109
USA
Tel: (858) 483-0024
Fax: (858) 483-0071

5.2 Composition of GL-ONC1 batches

Batch 004 05 08

Ingredients	One dose of 1.0 mL contains:	Function
Active substances		
Live recombinant LIVP strain GL-ONC1	1.22×10^8 pfu/mL	Active ingredient
Excipients		
Tris (hydroxymethyl)-amino methane, pH 7.7	1.21 mg	Component of dilution buffer
Sodium chloride	8.18 mg	Component of dilution buffer

Batch 006 09 11
Below is information for an example of an additional GL-ONC1 batch that may be used in this trial. Please

note, other batches may be used throughout the trial:

Ingredients	One dose of 1.0 mL contains	Function
**Active substances**		
Live recombinant LIVP strain GL-ONC1	1.74×10^8 pfu/mL	Active ingredient
**Excipients**		
Tris (hydroxymethyl)-amino methane, pH 7.7	1.21 mg	Component of dilution buffer
Sodium chloride	8.18 mg	Component of dilution buffer

5.3 Method of Preparation

The product is prepared by infecting chicken embryo fibroblasts (CEF) cells with the primary inoculum (for master seed virus: MSV) or with MSV (for working seed virus: WSV). After incubation, the product was harvested and refined through cell suspension, centrifugation and other processes. Tests performed before final release include:

- Sterility
- Titration of virus (potency assay)
- Mycoplasma (culture method)
- Mycobacteria
- Extraneous agents using chicks
- Extraneous agents in vitro
- Extraneous agents in vivo
- Endotoxin
- Appearance
- Gentamycin content
- Extractable volume
- Total protein content
- Abnormal toxicity
- pH
- Identity
- Inserted genes (protein)

5.4 Manufacturing

GL-ONC1 was produced in accordance with GLP and GMP by IDT Biologika GmbH (IDT).

Address:
Am Pharmapark
D-06861 Dessau-Roßlau
Germany
Phone: +49 (34901) 885-0
Fax: +49 (34901) 8855-323

5.5 Stability

5.5.1 Genetic identity of GL-ONC1 with GLV-1h68

Quality control of GMP-manufactured GL-ONC1, using PCR and sequence testing by MICROMUN GmbH, Walther-Rathenaustraße 49a, Biotechnikum Greifswald, 17489 Greifswald, Germany, has shown that there is some evidence for the presence of viral DNA with non-recombinant F14.5L-insertion region, but the amount of this part is regarded as minor. Sequence analysis demonstrated a minor population of viruses carrying a wild-type-like sequence in the F14.5L region. Viral plaque assays show populations where approximately 40% of the viruses express the diagnostic marker gene GFP from the F14.5L locus; the other diagnostic markers,

Galactosidase and Glucuronidase, are expressed 100% of the time. PCR and sequence analysis of 10 dark plaques isolated from the GMP batch indicated that silencing of GFP was mainly due to point mutations within both the promoter and coding regions, while only one plaque yielded wt F14.5L sequence. Both the manufacturing company, IDT, and Genelux have strong supporting data showing that changes this finding in the F14.5L region is a result of the virus' intent to silence the insert cassette to enhance its ability to propagate. We have also shown that using CEF cells, GFP fluorescence is already diminished after 3 passages and nearly lost after 6 passages, whereas material from the same virus aliquot showed more consistent GFP expression over more than 6 passages in CV-1 cells. These data are explained by faster growth and a significantly higher yield in CV-1 cells compared to CEF cells under laboratory conditions.

Nevertheless, this finding has no adverse effects as shown by the results of the toxicology and safety pharmacology testing in mice and rats using the clinical trial material by our contracted GLP animal facility. Furthermore, there is no impact on the tumor targeting or the oncolytic capacity (potency), as shown in data provided by the Genelux Research and Development division.

In order to analyze the impact of the absence of GFP expression, GLV-1h71 was constructed by replacing the *RUC-GFP* expression cassette in the genome of GLV-1h68 with a non-coding sequence. This virus showed equal therapeutic effects when compared with GLV-1h68 and was as safe as GLV-1h68 in nude mice bearing GI-101A xenografts. Thus, absence of GFP expression has no adverse influence on the safety and potency of GL-ONC1.

Risk of recombinations and deletions

Genomic differences among vaccinia virus strains are mainly due to variability within the terminal regions of the DNA molecule. The central part of the genome is highly conserved. Cell culture studies have demonstrated that functions coded within the DNA from the left-hand and right-hand terminal regions of the orthopoxvirus genome are not essential for replication of the virus, but rather for determination of host range and virulence.[14]

The risk that GL-ONC1 will revert into more virulent mutants is very low, as the parental LIVP strain, a descendant of the Lister strain, was attenuated by frequent passages on calf skin (more than 500 times) and was widely used as a vaccine, with excellent documented safety, during the WHO smallpox eradication program. In addition, because orthopoxviruses are not endemic in the human population, it is unlikely that GL-ONC1, as a clonal strain, will recombine with a wild-type orthopoxvirus to produce a more virulent strain; and, despite worldwide use of the live virus vaccine, no reported adverse events due to mutation to a more aggressive phenotype has ever been reported. Various strains of vaccinia virus (such as NYCBH) could be detected in humans shortly after receiving vaccinia-based vaccines.

Recombination of orthopoxviruses with other DNA/RNA-viruses has been reported very rarely and appears to be very unusual. In addition, since poxviruses use unique promoters, gene activation by inserted foreign promoters also seems very unlikely.

Physical Stability of GL-ONC1

Samples of GLP (starting titer: 3.92×10^8 pfu/mL) and GMP (starting titer: 6.17×10^7 pfu/mL) manufactured GL-ONC1 were spotted onto filter paper to examine the stability after release into environment. At the respective time points, the virus samples were retrieved from the filter paper and the amount of active virus was analysed by plaque assay.

Our experimental results showed that the titres of both GLP and GMP manufactured GL-ONC1 were decreased by 99.99% within 24 hrs when released into the environment at room temperature. By days 6-7, all

viruses were disintegrate. Therefore, we do not believe that shedded virus from patients, if any, will be of significant environmental concerns or health concerns to the others.

Ability of GL-ONC1 after release into environment

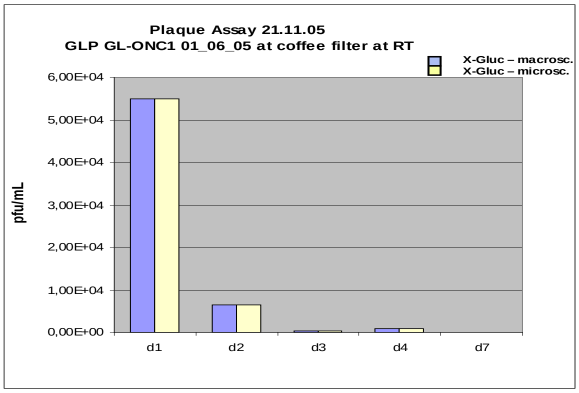

A) GLP GL-ONC1 - Starting titer: 3,92 E+08 pfu/ml

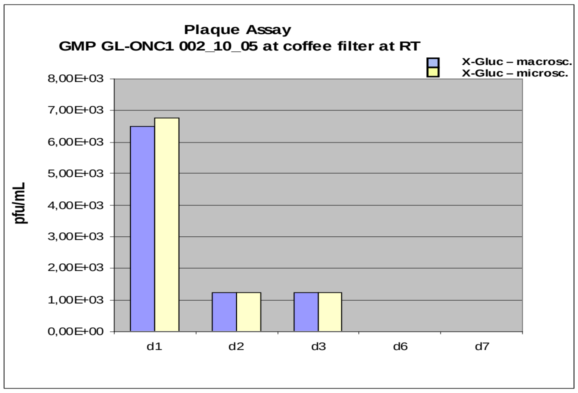

B) GMP GL-ONC1 - Starting titer: 6,17E+07 pfu/ml

The sponsor will supply the principal investigator with GL-ONC1 packaged in glass vials as a 1.2 mL aqueous suspension. Vial labels will indicate the product, lot number and concentration. (pfu/mL). The vials will be stored at -70°C ±10ºC until they are thawed and stored on ice or at 2-8°C in preparation for dilution. Each vial should be vortexed at full power for 1 minute prior to preparation for dilution (full details of the supply of GL-ONC1 and the requirements for dispensing will be provided in the Drug Handling Guidelines (DHG)). The procedure will be undertaken in a closed room with appropriate precautions including mask, gown and gloves. All material that comes into contact with the viral preparation will be autoclaved. GL-ONC1 will be administered as a bolus injection.

The following information will be listed on each vial label:

- Product: GL-ONC1
- Study #: GL-ONC1-003/MSKCC
- Agent: GL-ONC1 (1.22 x 10^8 pfu/mL)
- Volume: 1.0 mL
- Lot Number: 004 05 08, Vial No. xxxx
- Expiry Date:
- For Intrapleural Infusion
- For Clinical trial Use Only

The label affixed to the finished box will contain the following components:

- Product: GL-ONC1
- Caution: New Drug--Limited by Federal (or United States) law to investigational use.
- Study #: GL-ONC1-003/MSKCC
- IND #: BB-IND 15234
- Sponsor: Genelux GmbH, Am Neuland 1, 82347 Bernried, Germany, Phone: +49 8158-9223-20
- Manufactured for Genelux GmbH by: IDT Biologika GmbH, Am Pharmapark D-06861 Dessau-Roßlau Germany Phone: +49 34901-885-0
- Agent Concentration: 1 vial contains 1.22 x 10^8 pfu/mL of GL-ONC1
- Contents: 6 vials of Lot # 004 05 08
- Expiry Date:
- Store at -70°C ± 10°C
- Sterile suspension for intrapleural infusion
- Box #:
- Prepare and administer according to directions provided

- Principal Investigator: Valerie Rusch, M.D.

For detailed instruction on the preparation of GL-0NC1 for administration, refer to the Drug Handling Guideline (DHG) which will be available at all times to personnel involved in the study. The pharmaceutical dosage form and quantity of dosage units may also be omitted from the label of the primary packaging; however, detailed instruction will be provided in the DHG for the preparation of GL-ONC1 for administration as a 500 mL bolus injection.

Storage and handling

Package quantity:	Six vials per carton
Nominal batch size:	**Batch 004 05 08**<br>3428 vials with 1.22×10^8 pfu/mL<br><br>**Batch 006 09 11**<br>843 vials with 1.74×10^8 pfu/ mL
Appearance:	Pale, milky to light brown-colored homogenous suspension
Storage:	-70 ºC ±10ºC
Dosage form:	Aqueous suspension
Container:	2-mL injection vials, 2R, made of clear borosilicate glass, FIOLAX®-klar, WKB 1, KIN ISO 8362/1. The vials are supplied by Thüringer, Pharmaglass, Germany. The vials are labeled with printed self-adhesive paper label.
Closure system:	13 mm rubber injection stoppers, FM 257/2, V 9218, SAF 1, DIN ISO 8362-5-13. Supplied by Helveot Pharma, Belgium.<br>13 mm aluminum caps, gold, DIN ISO 8362/3. Supplied by BICO Pharma Verpackungen GmbH.
Expiration:	Two years, regular checks for viability. The expiration date may be extended in agreement with the sponsor by conducting stability analyses.

Preparation for infusion

The dilution to a final PFU must be performed in a sterile hood under Biosafety level 2 conditions by the principal investigator or qualified designated study personnel:

1. Remove vial from storage.
2. Thaw; Keep on ice or at 2-8°C until use (no more than 2 hr).
3. Vortex at full power for 1 minute immediately prior to dilution.
4. Refer to the Drug Handling Guideline for detailed descriptions on the preparation of the virus dilutions for each cohort.
5. Transfer the solution completely into a 500 mL infusion bag:
   a. Discharge the pleural fluid (500 mL),
6. Infuse 500 mL Ringer's Lactate solution intrapleurally via the small lumen of the trocar catheter.
7. Administer the virus solution in the infusion bag as a bolus via the trocar catheter.
8. After virus delivery, infuse 10 mL Ringer's Lactate solution via the trocar catheter to clean the virus from the tube.
9. All material that comes in contact with the viral preparation will be autoclaved.

Further precise details for preparation and dilution to a final pfu dose are found in the Drug Handling Guideline.

Drug Accountability

Once the proper permits have been documented, the manufacturer will provide the principal investigator with sufficient amounts of the investigational product, GL-ONC1. The principal investigator or designee will confirm receipt of all batches of GL-ONC1 in writing.

The principal investigator/investigator will administer GL-ONC1 only to qualified patients who provide documented written consent to participate in this study, and according to the procedures set out in this study protocol. Each administration of GL-ONC1 will be documented in the CRF.

All supplies must be accounted for throughout the trial and at the end of the study. The principal investigator or designee must maintain accurate and adequate records including dates, lot numbers, quantity received, individual usage, destroyed product, etc. The principal investigator or designee may return unused supplies to the manufacturer, noting the exact amount used in the study, regardless of whether the study was completed or terminated prematurely, or with the manufacturer's concurrence, the principal investigator or designee may destroy unused product on site according to the institution's disposal practices for biologic material. If the principal investigator elects to return unused study drug, the principal investigator or designee must verify that all unused or partially used study drug supplies have been returned and that no remaining supplies are in their possession.

6.0 CRITERIA FOR SUBJECT ELIGIBILITY

Describe the characteristics of the patient/subject population.

6.1 Subject Inclusion Criteria

Inclusion Criteria

- Diagnosis of histologically or cytologically documented, malignant pleural effusions (primary non-small-cell lung carcinoma, mesothelioma, and other histologies), who have free pleural space (partial or total) that permits the intrapleural drug instillation. This includes cytologically negative pleural effusion in conjunction with histologically proven malignancy involving the pleura.
- Age must be ≥ 18 years.
- All acute toxic effects of any prior radiotherapy, chemotherapy, or surgical procedures must have resolved to Common Terminology Criteria for Adverse Events (CTCAE, Version 4.0) Grade ≤ 1.
- Any surgery, where general anesthesia was administered, must have occurred at least 7 days prior to study enrollment.
- Chemotherapy, radiotherapy or immunotherapy must have stopped more than 14 days prior to receiving study drug; however, small field palliative radiotherapy, TKI therapies, and hormonal therapies are allowed.
- Patients with stage IV malignancy (non-mesothelioma)must have had a brain scan (MRI or CT with contrast) showing no evidence of disease progression within 8 weeks of study enrollment.
- EGOG/Zubrod ≤ 2.
- Required baseline laboratory data include
  - Absolute neutrophil count (ANC) ≥ 1.5 × 10^9 [SI units 10^9/L],
  - Platelets ≥ 100 ×10^9 [SI units 10^9/L],
  - Hemoglobin ≥ 9.0 g/dL [SI units gm/L],
  - Serum creatinine ≤ 1.5 × upper limit of normal (ULN),
  - Bilirubin ≤ 1.5 × ULN,
  - AST/ALT ≤ 2.5 × ULN (≤ 5 × ULN in the presence of liver metastases)
    Negative pregnancy test for females of childbearing potential.

6.2 Subject Exclusion Criteria

Exclusion criteria

- Pregnant or breast-feeding women.
- Patients with fever or any active systemic infections, including known HIV, hepatitis B or C.
- Patients on immunosuppressive therapy or with immune system disorders, including autoimmune diseases.
- Concurrent steroid use of more than an equivalent of 20 mg/day prednisone (or equivalent).
- Prior splenectomy.
- Previous organ transplant.
- Patients with clinically significant dermatological disorders, e.g., eczema or psoriasis, as judged by the principal investigator, or any unhealed skin wounds or ulcers.
- Clinically significant cardiac disease (New York Heart Association, Class III or IV).
- Dementia or altered mental status that would prohibit informed consent.
- Other severe, acute, or chronic medical or psychiatric condition or laboratory abnormality, that may increase the risk associated with study participation or study drug administration, or may interfere with the interpretation of study results and, in the judgment of the principal investigator, would make the patient inappropriate for this study.
- Known allergy to ovalbumin or other egg products.
- Prior gene therapy treatments or prior therapy with cytolytic virus of any type.
- Concurrent therapy with any other investigational anticancer agent.
- Concurrent antiviral agent active against vaccinia virus (e.g. cidofovir, vaccinia immunoglobulin) during the study.

7.0 RECRUITMENT PLAN

Potential research subjects will be identified by a member of the patient's treatment team, the principal investigator, or research team at Memorial Sloan-Kettering Cancer Center (MSKCC). If the principal investigator is a member of the treatment team, s/he will screen their patient's medical records for suitable research study participants and discuss the study and their potential for enrolling in the research study. Potential subjects contacted by their treating physician will be referred to the principal investigator/research staff to enroll in the study, and to record appropriate contact information in order to approach these patients regarding the possibility of enrolling in the study staff of the study.

The principal investigator/research staff may also screen the medical records of patients with whom they do not have a treatment relationship for the limited purpose of identifying patients who would be eligible to enroll in the study and to record appropriate contact information in order to approach these patients regarding the possibility of enrolling in the study.

During the initial conversation between the principal investigator/research staff and the patient, the patient may be asked to provide certain health information that is necessary to the recruitment and enrollment process. The principal investigator/research staff may also review portions of their medical records at MSKCC in order to further assess eligibility. They will use the information provided by the patient and/or medical record to confirm that the patient is eligible and to contact the patient regarding study enrollment. If the patient turns out to be ineligible for the research study, or declines to participate, the research staff will destroy all information collected on the patient during the initial conversation and medical records review, except for any information that must be maintained for screening log purposes.

In most cases, the initial contact with the prospective subject will be conducted either by the treatment team, investigator or the research staff working in consultation with the treatment team. The recruitment process outlined presents no more than minimal risk to the privacy of the patients who are screened and minimal PHI

will be maintained as part of a screening log. For these reasons, we seek a (partial) limited waiver of authorization for the purposes of (1) reviewing medical records to identify potential research subjects and obtain information relevant to the enrollment process; (2) conversing with patients regarding possible enrollment; (3) handling of PHI contained within those records and provided by the potential subjects; and (4) maintaining information in a screening log of patients approached (if applicable).

8.0 PRETREATMENT EVALUATION

The schema for this protocol for study intervention is included as Appendix 1.

Prior to study enrollment each patient will have the following assessments.

Within 14 days of protocol treatment:

Routine tests/procedures:

- Provision of informed consent by patient.
- Tumor evaluation by chest CT. Optimally this will be done after drainage of the pleural space unless for reasons of clinical safety a CTis required to characterize the location and extent of the effusion prior to drainage.
- Medical history and physical examination.
- Assessment of concomitant medications.
- Vital signs (blood pressure, pulse rate and temperature).
- 12-lead ECG.
- Complete Blood Count (CBC) & Comprehensive Metabolic Panel, LDH, & SMRP (only in mesothelioma patients).
  Serum pregnancy test (if applicable).

Experimental tests/procedures:

- Anti-vaccinia virus neutralizing antibodies titers.
- C-Reactive Protein
- Immunophenotyping of lymphocytes and cytokines analysis in blood (prior to treatment). Analysis of lymphocyte subsets (e.g. CD4, CD8, CD19, CD25, CD56, CD69) and cytokine profile (plasma-INF gamma, TNF and IL-1)

Within 8 weeks of study treatment:

Routine tests/procedures:

- Patients with stage IV malignancy (non mesothelioma); a brain scan (MRI or CT with contrast) showing no evidence of disease progression.

9.0 TREATMENT/INTERVENTION PLAN

The pleural effusion will be drained via insertion of a chest tube or PleurX catheter. A chest CT scan will be performed, optimally after drainage of the effusion to assess the extent of pleural disease. The drainage and CT scan can be performed on an outpatient basis or following admission to the hospital for treatment on protocol. The chest CT scan will be done within 14 days of planned therapy start date. Instillation of the virus will occur after admission to the hospital. The pleural space will be drained prior to instillation of virus. Pleural fluid is then processed as instructed in the *Biological Sample and Handling Manual* and frozen at -70°C. After drainage of the pleural space via the chest tube/catheter, 250 ml of Ringer's Lactate will be infused and then

drained. The virus will be instilled as a bolus into the pleural space via the chest tube or pleural catheter. Up to 150 ml of additional saline may be used to flush the chest tube/PleurX catheter to ensure that the entire treatment drug reaches the pleural space. The chest tube/PleurX catheter will be left clamped for 4 hours (+/- 1 hour) after which it will be reopened in order to drain the pleural space. As dictated by the patient's clinical status, the chest tube/PleurX may either be left in or removed at the VATS procedure performed 2-9 days after treatment.

Virus administration:

Table: Dose level by cohort (flat dose independent of body weight)

Cohort	Dose **	Number of doses	Total volume of each injection
-1*	1×10^6	1	Final volume of preparation will be 500 mL, to be administered as a bolus.
+1	1×10^7	1	
+2	1×10^8	1	
+3	1×10^9	1	
+4	3×10^9	1	
+5***	3×10^9	3	
+6***	6×10^9	3	
+7***	1×10^{10}	3	
Phase IIa*** (expansion)	(selected dose)	3	

* Necessary only if a DLT is encountered at the initial dose level.
** Dose de-escalation, if needed, will be done to intermediate levels that are one-half log lower, unless DSMC advises for full dose de-escalation
*** The dose of cohorts 5 or 6 or 7 may be selected as the expansion cohort based on safety. Three additional patients will be treated at the selected dose for expansion.

Antidote – Rescue Medication

Patients who develop serious harmful side effects due to viral administration will be immediately treated by the principal investigator or designee. Since GL-ONC1 is a replication-competent virus, it may cause generalized vaccinia infection. The medication, vaccinia immunoglobulin (VIG) (or equivalent), will be available for use in case of a severe toxicity related to this GL-ONC1.

It is preferable to avoid the use of corticosteroids unless the treating physician believes it is absolutely necessary for the safety of the patient. Anti-vaccinia therapy will only be used if there is evidence of a systemic vaccinia infection, according to Appendix 3 "Treatment Guidelines for use of Vaccinia immunoglobulin (VIG)".

Concomitant therapy

All concomitant medications must be recorded in the patient's Case Report Form (CRF). No surgery, investigational treatment, radiotherapy (other than small-field palliative radiotherapy), chemotherapy, immunosuppressants or immunotherapy is allowed during the course of this study. Routine prophylactic use of growth factor (G-CSF or GM-CSF) is not allowed. Concurrent corticosteroid usage is not allowed if more than a 20 mg per day prednisone (or equivalent) is applied.

Hormonal therapy with LHRH analogues for patients with prostate cancer is permitted in the face of a rising PSA. Concurrent bisphosphonates are also allowed. The use of erythropoietin or blood transfusions is permitted at the discretion of the treating physician. Anticoagulant therapy is also permitted. Apart from the excluded concomitant therapies listed above, other concomitant medications can be administered at the discretion of the principal investigator.

Management of toxicity

The following toxicities experienced by protocol patients will be managed accordingly:

1. Flu-like symptoms (fever, headache, myalgia, arthralgia): these will be treated with aspirin, acetaminophen, equivalent NSAID (non-steroidal anti-inflammatory drugs) or antihistamines as indicated.
2. Diarrhea: this will be treated with imodium or loperamide or equivalent.
3. Nausea and vomiting: these will be treated with metoclopramide and/or 5HT3 receptor antagonists or equivalent.
4. Rash: instruct the patient to keep the area clean and not scratch the rash

10.0 EVALUATION DURING TREATMENT/INTERVENTION

The schema for this protocol with study intervention is included in Appendix 1.

Routine tests/procedures:

- Medical history and physical exam (at PO clinical follow-up visit (between Day 14 and 30), , and at the end of the study visit (day 60±10)).
- Assessment of concomitant medications (on Day 1 prior to treatment, PO clinical follow-up visit (between Day 14 and 30, and at the end of the study visit (day 60±10)).
- Evaluation of performance status (ECOG/Zubrod): (Day 1 prior to treatment , Days 2, 3, 4, & 5, PO clinical follow-up visit (between Day 14 and 30), and at the end of the study visit (day 60±10)).
- CBC, COMP, LDH, and SMRP (only mesothelioma patients) (Days 2, 3, 4 & 5, PO clinical follow-up visit between days 14-30, and at the end of the study visit (day 60±10).
- Vital signs: blood pressure, pulse rate and temperature (Day 1 prior to initial treatment, prior to each subsequent treatment, 30(±15) minutes, 60(±15) minutes, 120(±15) minutes, 8(±1) hours, 16(±1) hours post each treatment, , at the PO clinical follow-up visit between days 14 – 30, and at the Day 60(± 10) follow up visit).
- Tumor evaluation by chest CT after drainage of the pleural space (if necessary) at end of study (day 60±10 follow up).

Experimental tests/procedures:

- Assessment of adverse events: Toxicity assessment (Day 1 prior to initial treatment, prior to each subsequent treatment, 30(±15) minutes, 60(±15) minutes, 120(±15) minutes, 8(±1) hours, 16(±1) hours post each treatment, each subsequent inpatient day,PO clinical follow up visit between days 14-30, and at the Day 60(±10) follow up visit.
- Video-Assisted Thoracoscopic Surgery (VATS) for GFP imaging (Days 2 -9)):
  For those patients who have a need for operative pleurodesis and it is clinically indicated, the patients will have assessment of tumor infection by operative assessment for green fluorescent protein (GFP) expression in tumor and surrounding tissues. Photographic and videographic documentation of fluorescence may be performed. Patients may also have random biopsies and GFP-directed biopsies performed to allow for assessment of viral presence (if applicable). Viral plaque assays (VPA) will be performed in tumor biopsies Immunohistochemical (IHC) staining for GL-ONC1 and beta-glucuronidase testing will be performed on both GFP (-) and (+) areas at videothoracoscopy (if applicable). For patients who have a medical contraindication to VATS, pleural biopsies will not be performed but other assays documenting viral presences will be performed.

- Viral detection: (Prior to initial treatment, Days 2, 3, 4, & 5) Sampling of blood, sputum, urine, and pleural fluid for evaluation of viral particles by VPA. *If a patient is unable to physically produce a sample (urine, blood, sputum, and/or pleural fluid) this will not constitute a protocol violation.*
- C-Reactive Protein (Days 2, 3, 4 & 5, PO clinical follow-up visit between days 14-30, and at the end of the study visit (day 60±10 days).
- Anti-vaccinia virus neutralizing antibodies titers (day 60±10 days; i.e. at the end of the study).
- Immunophenotyping of lymphocytes and cytokines analysis in blood (prior to initial treatment, and Days 2 and 4, and day 60 (± 10 days)). Analysis of lymphocyte subsets (e.g., CD4, CD8, CD19, CD25, CD56, CD69) and cytokine profile (plasma-IFN gamma, TNF and IL-1) will be performed.

Premature termination of the study

If the principal investigator or designee becomes aware of conditions or events that suggest a possible hazard to patients, they must notify the IRB immediately.

The study may be terminated early at the principal investigator's discretion in the absence of such findings. Conditions that may warrant termination include, but are not limited to:

- The discovery of an unexpected, significant, or unacceptable risk to the patients enrolled in the study.
- Unsafe or unethical practices.
- Failure of the principal investigator to enter patients at an acceptable rate.
- A decision on the part of the principal investigator to suspend or discontinue the development of the program.

Clinical Follow-up Visits: These visits are conducted between days 14-30 and 60±10 days as a clinical evaluation of patient's progress following treatment.

Procedures include:
- Medical history.
- Physical exam.
- Vital signs.
- Concomitant medication.
- EGOG/Zubrod performance status.
- Collection of adverse event toxicity information.
- CBC, Comprehensive metabolic panel, LDH & SMRP (mesothelioma patients only)
- Research bloodwork
- Chest CT scan

Follow up

After the patient discontinues the treatment phase (i.e. 60±10 days after receiving virus treatment(s)), the principal investigator or designee will make every effort to continue to evaluate each patient for delayed toxicity by clinical and laboratory evaluations as clinically indicated.

Post day 60 (+/-10) CT scans obtained as routine clinical management of patient may be included to follow disease status of patient (e.g., PFS).

As required by federal regulations, there will be periodic subsequent phone assessments for status of patients until death. The phone calls will approximately take place every 6 to 12 months if not being followed clinically by MD at MSK.

11.0 TOXICITIES/SIDE EFFECTS

Safety evaluations:

The safety of GL-ONC1 will be assessed by the evaluation of the type, frequency, and severity of adverse events (AEs), changes in clinical laboratory tests (hematological and chemistry), immunogenicity and physical examination. All AEs and laboratory toxicities will be graded on the Common Terminology Criteria for Adverse Events (CTCAE version 4).

Management of toxicity

The following toxicities from GL-ONC1 experienced by patients will be managed accordingly:

1. Flu-like symptoms (fever, headache, myalgia, arthralgia): these will be treated with aspirin, acetaminophen, equivalent NSAID (non-steroidal anti-inflammatory drugs) or antihistamines as indicated
2. Diarrhea: this will be treated with imodium or loperamide or equivalent
3. Nausea and vomiting: these will be treated with metoclopramide and/or 5HT3 receptor antagonists or equivalent
4. Rash: instruct the patient to keep the area clean and not to scratch the rash

12.0 CRITERIA FOR THERAPEUTIC RESPONSE/OUTCOME ASSESSMENT

Assessment of Antineoplastic Activity

Although response is not the primary endpoint of this trial, patients with measurable disease will be assessed by RECIST criteria and modified RECIST for malignant mesothelioma. For the purpose of this trial, patients will be evaluated clinically and radiologically before and after treatment as shown in the study calendar. Response and progression will be evaluated using internationally accepted response criteria and definitions. For solid tumors the international criteria proposed by the Response Evaluation Criteria in Solid Tumors (RECIST) Committee (Therasse *et al*. 2000) will be used as described below:

Definitions

Changes in only the largest diameter (unidimensional measurement) of the tumor lesions will be used in the RECIST criteria. Note: Lesions are either measurable or non-measurable using the criteria provided below. The term “evaluable” in reference to measurability will not be used because it does not provide additional meaning or accuracy.

Measurable Disease

Measurable lesions are defined as those that can be accurately measured in at least one dimension (longest diameter to be recorded) as ≥20 mm with conventional techniques (PET, CT, MRI, x-ray) or as ≥10 mm with spiral CT scan. All tumor measurements must be recorded in millimeters (or decimal fractions of centimeters).

Non-measurable Disease

All other lesions (or sites of disease), including small lesions (longest diameter <20 mm with conventional techniques or <10 mm using spiral CT scan), are considered non-measurable disease. Bone lesions,

leptomeningeal disease, ascites, pleural/pericardial effusions, lymphangitis cutis/pulmonis, inflammatory breast disease, abdominal masses (not followed by CT or MRI), and cystic lesions are all non-measurable.

Target Lesions

All measurable lesions up to a maximum of five lesions per organ and 10 lesions in total representative of all involved organs should be identified as target lesions and recorded and measured at baseline. Target lesions should be selected on the basis of their size (lesions with the longest diameter) and their suitability for accurate repeated measurements (either by imaging techniques or clinically). A sum of the longest diameter (LD) for all target lesions will be calculated and reported as the baseline sum LD. The baseline sum LD will be used as the reference by which to characterize the objective tumor response.

Non-target Lesions

All other lesions (or sites of disease) should be identified as non-target lesions and should also be recorded at baseline. Non-target lesions include measurable lesions that exceed the maximum numbers per organ, or total of all involved organs as well as non-measurable lesions. Measurements of these lesions are not required but the presence or absence of each should be noted throughout follow-up.

Guidelines for Evaluation of Measurable Disease

All measurements should be taken and recorded in metric notation using a ruler or calipers. All baseline evaluations should be performed as closely as possible to the beginning of treatment and never more than 4 weeks before the beginning of the treatment.

Note: Tumor lesions that are situated in a previously irradiated area might or might not be considered measurable.

The same method of assessment and the same technique should be used to characterize each identified and reported lesion at baseline and during follow-up. Imaging-based evaluation is preferred to evaluation by clinical examination when both methods have been used to assess the antitumor effect of a treatment.

Clinical Lesions:

Clinical lesions will only be considered measurable when they are superficial (e.g., skin nodules and palpable lymph nodes). In the case of skin lesions, documentation by color photography, including a ruler to estimate the size of the lesion, is recommended.

Chest X-ray:

Lesions on chest x-ray are acceptable as measurable lesions when they are clearly defined and surrounded by aerated lung. However, CT is preferable.

Conventional CT

These techniques should be performed with cuts of 10 mm or less in slice thickness contiguously. Spiral CT should be performed using a 5 mm contiguous reconstruction algorithm. This applies to tumors of the chest, abdomen, and pelvis. Head and neck tumors and those of extremities usually require specific protocols.

Response Criteria

Evaluation of Target Lesions

Complete Response (CR): Disappearance of all target lesions.

Partial Response (PR): At least a 30% decrease in the sum of the longest diameter (LD) of target lesions, taking as reference the baseline sum LD.

Progressive Disease (PD): At least a 20% increase in the sum of the LD of target lesions, taking as reference the smallest sum LD recorded since the treatment started or the appearance of one or more new lesions.

Stable Disease (SD): Neither sufficient shrinkage to qualify for PR nor sufficient increase to qualify for PD, taking as reference the smallest sum LD since the treatment started.

Evaluation of Non-Target Lesions

Complete Response (CR): Disappearance of all non-target lesions and normalization of tumor marker level.

Incomplete response / Stable Disease (SD): Persistence of one or more non-target lesion(s) or/and maintenance of tumor marker level above the normal limits.

Progressive Disease (PD): Appearance of one or more new lesions and/or unequivocal progression of existing non-target lesions.

Although a clear progression of “non-target” lesions only is exceptional, in such circumstances, the opinion of the treating physician should prevail, and the progression status should be confirmed at a later time by the review panel (or trial chair).

Note: If tumor markers are initially above the upper normal limit, they must normalize for a patient to be considered in complete clinical response.

Evaluation of Best Overall Response

The best overall response is the best response recorded from the start of the treatment until disease progression/recurrence (taking as reference for progressive disease the smallest measurements recorded since the treatment started). The patient's best response assignment will depend on the prior mentioned response criteria.

Confirmatory Measurement/Duration of Response

Duration of Overall Response

The duration of overall response is measured from the time measurement criteria are met for CR or PR (whichever is first recorded), until the first date that recurrent or progressive disease is objectively documented (taking as reference for progressive disease the smallest measurements recorded since the treatment started).

Duration of Stable Disease

Stable disease is measured from the start of the treatment until the criteria for progression are met, taking as reference the smallest measurements recorded since the treatment started.

Duration of Response

Duration of response is defined as the time of first notation of response until the time of first notation of disease progression.

Modified RECIST criteria

In response to the difficulties to reproducibly measure tumor response to treatment in patients diagnosed with malignant pleural mesothelioma, Byrne and Nowak[15] developed a modified RECIST criteria for patients with this diagnosis. Due to the growth pattern of malignant mesothelioma around the pleural surface, the standard RECIST criteria does not take into account the difficulties inherent in the placement of the longest unidimensional measurements, as well as comparison with bidimensional disease.

Measurement Procedures

The tumor thickness that is perpendicular to the chest wall or mediastinum is measured in two positions at three separate levels on transverse cuts of CT scan. The sum of these six measurements provide a pleural unidimensional measurement. Transverse cuts should be taken at least 1 cm apart and relate to anatomical landmarks in the thorax for reproducible assessment at later imaging time points. If measureable tumour is present, transverse cuts in the upper thorax, above the level of division of the main bronchi are preferred. At reassessment, pleural thickness is measured at the same position, and at the same level as previous imaging time points. Nodal, and other measurable lesions should be measured as indicated above for RECIST criteria. To obtain the total tumor measurement, add up all unidimensional measurements and refer to the evaluation of response criteria listed above.

13.0 CRITERIA FOR REMOVAL FROM STUDY

If at anytime the patient is found to be ineligible for the protocol as designated in the section on Criteria for Patient/Subject Eligibility (i.e., a change in diagnosis), the patient will be removed from the study, unless the ineligibility could be corrected with supplemental therapies, or under the discretion of the principal investigator, the patient is still determined to be suitable for the study.

The principal investigator/research staff will maintain a list of all patients who sign the informed consent form. Patients who sign the informed consent form but fail to meet one or more of the eligibility criteria, or who decline study participation before receiving the study drug, are defined as screen failures. Reasons for ineligibility will be documented in the screening log.

Patients will be removed from the study assessments if any of the following events occur:

1. Patient refusal: Patients may withdraw their consent to participate in the study at any time without prejudice. Patients are not required to state any reasons for withdrawing consent.
2. Patient lost to follow-up: The principal investigator and study staff are expected to attempt to contact any patient who is lost to follow-up, and to document such contacts in the source documents

Every effort must be made to contact the physicians providing ongoing care for study participants to determine whether adverse events occur and to obtain a date of death, if applicable. This is in order to maximize the safety data obtained from the trial and therefore maximize the safety of future patients treated with GL-ONC1.

Patients may also be required to withdraw after discussion with principal investigator or designee for the following reasons:

- Adverse events
- Deviation from the treatment plan specified in the protocol (e.g. incorrect administration of study drug or

treatment)
- Patient non-compliance (e.g., failure to attend study visits)

Any patient who withdraws from study treatment should return for safety follow-up visits. Patients who withdraw from the study after receiving the drug will be followed up post-study, along with the rest of the patients.

The reason(s) for withdrawal must be recorded in the Case Report Form (CRF) and in the patient's medical record. If there is more than one reason for withdrawal from the study/treatment, one reason will be shown in the CRF as the primary reason and others will be shown as secondary reasons. In case of loss to follow-up, an effort must be made to determine why a patient fails to return to the clinic for the study visits. Patients who withdraw consent will have the reason documented as "withdrawal of consent".

Patients not receiving the treatment for any reason will be replaced.

14.0 BIOSTATISTICS

Thirteen patients (12 evaluable) have been treated thus far with the initial 3+3 dosing escalation schema. No DLT have been experienced MTD has not been reached. One additional patient is in treatment as part of the original planned expansion cohort at level D4 and no treatment related SAEs have occurred. Thus, we plan recommend transitioning to consecutive daily dosing to GL-ONC1 for up to 3 days.

DLT criteria are described in Section 4.3. In order to determine the maximum tolerated dose (MTD), a modified 3+3 escalation dose schema will be used. Seven doses are now included in the escalation scheme, with one additional dose reserved for de-escalation if dose level 1 proves to be too toxic. In addition, if de-escalation from doses 2 through 7 is needed, patients will be treated at intermediate doses half-log lower that the dose from which the de-escalation starts, unless DSMC advises for full dose de-escalation, The dose escalation scheme is as follows:

(1) If none of the initial three patients at a given dose level experience DLT, the next dose level will be studied.
(2) If one of the initial three patients at a given dose level experiences DLT with a determination of relatedness to GL-ONC1, three additional patients will be treated at the same dose level. Escalation will continue only if there has been no additional DLT observed.
(3) If two or more patients experience DLT with a determination of relatedness to GL-ONC1, it is considered that DLT for that cohort has been reached. Then, an intermediate dose level of one-half log lower dose will be studied in 3-6 patients, and, if tolerated, it will be the MTD. Per DSMC advice, de-escalation can be done to a full dose.
(4) If only three patients were treated at a dose under consideration as MTD, an additional three patients will be treated at that level to confirm previous results

Once the MTD is determined, a maximum of 3 additional patients will be treated at the MTD level (for a total of 6 patients) to better define tolerability, viral replication and pharmacodynamics. If an excessive number of DLTs will be observed in the expansion cohort, the MTD will be rediscussed.

A patient is considered toxicity-free for the purpose of the trial if he/she does not experience a dose limiting toxicity (DLT) within the 14 days following vaccine administration. No within-patient escalation will be performed. Patients who cannot undergo 14 days of evaluation for any reason other than experiencing DLT will be replaced.

The dose escalation-de-escalation scheme provides the following probabilities of escalation based on the true chances of DLT at a specific dose level. One can see that the probability of escalation is high if the toxicity risks are low.

True Risk of Toxicity	10%	20%	30%	40%	50%	60%
Probability of Escalation	91%	71%	49%	31%	17%	8%

A minimum of 4 patients will be enrolled. Maximum number of patients depends on the number of doses actually tested and number of DLTs encountered at each dose. If no de-escalation is necessary, the maximum number of evaluable patients will be 25 (12 patients already evaluated at D1-D4 at the time of this amendment, plus one patient in treatment in the originally planned dose expansion cohort at D4, plus a maximum of 3x3 = 9 patients at D5-D7, plus 3 additional patients in the expansion cohort). Considering possible de-escalation at intermediate doses, the maximum number of evaluable patients will be 34. With an expected accrual rate of 2 patients/month, we expect the study to conclude accrual within 12-18 months.

Safety data will be assessed in terms of AEs, laboratory data and vital sign data, which will be collected for all patients. Appropriate summaries of these data will be presented. AE will be listed individually per patient according to CTCAE version 4.0.

The following secondary endpoints will be investigated for each vaccine dose and summarized using descriptive statistics:

- viral detection in blood, sputum, urine, and pleural fluid samples: serially collected pretreatment, Days 2, 3, 4, & 5.
- Beta-glucuronidase testing measures in the pleural fluid collected; serially collected pretreatment, Days 2, 3, 4, & 5.
- viral infection of the tumor, as documented through GFP expression and confirmed through VPA, immunohistochemical (IHC) staining and beta-glucuronidase testing measures in the biopsy tissue collected once, at Day 2-9 post-treatment;
- anti-vaccinia virus neutralizing antibody titres: measured in blood collected pretreatment and at the end of the study (Day 60±10 days post-treatment);
- lymphocytes and cytokine profiles: measured in blood collected pretreatment, Day 2 and Day 4, and at the end of the study (Day 60±10 days).

Therapeutic efficacy will be investigated with CT scans pretreatment and at Day 60 (+/-10) posttreatment. Response by RECIST criteria (and by modified RECIST – for mesothelioma tumors) will be summarized for each dose level using descriptive statistics.

15.0 RESEARCH PARTICIPANT REGISTRATION AND RANDOMIZATION PROCEDURES

15.1 Research Participant Registration

Confirm eligibility as defined in the section entitled Criteria for Patient/Subject Eligibility.

Obtain informed consent, by following procedures defined in section entitled Informed Consent Procedures.

During the registration process registering individuals will be required to complete a protocol specific Eligibility Checklist.

All participants must be registered through the Protocol Participant Registration (PPR) Office at Memorial Sloan-Kettering Cancer Center. PPR is available Monday through Friday from 8:30am – 5:30pm at 646-735-8000. Registrations must be submitted via the PPR Electronic Registration System (http://ppr/). The completed signature page of the written consent/RA or verbal script/RA, a completed Eligibility Checklist and other relevant documents must be uploaded via the PPR Electronic Registration System.

15.2 Randomization

This protocol does not entail patient randomization. All patients will receive the same treatment (at different dose levels depending on the cohort) throughout the protocol course.

16.0 DATA MANAGEMENT ISSUES

A Research Study Assistant (RSA) will be assigned to the study. The responsibilities of the RSA include project compliance, data collection, abstraction and entry, data reporting, regulatory monitoring, problem resolution and prioritization, and coordinate the activities of the protocol study team.

The data collected for this study will be entered into a secure database (CRDB) and also into the CRFs. Source documentation will be available to support the computerized patient record and for the purposed of source verification by the RSA assigned to study.

16.1 Quality Assurance

Weekly registration reports will be generated to monitor patient accruals and completeness of registration data. Routine data quality reports will be generated to assess missing data and inconsistencies. Accrual rates and extent and accuracy of evaluations and follow-up will be monitored periodically throughout the study period and potential problems will be brought to the attention of the study team for discussion and action.

Random-sample data quality and protocol compliance audits will be conducted by the study team, at a minimum of two times per year, or more frequently, if indicated.

This study will be conducted in accordance with this protocol, FDA regulations, the ICH Guidelines for Good Clinical Practice and the Declaration of Helsinki. The study will also be performed according to local legal requirements. The principal investigator must comply with all applicable regulations, local and institutional requirements and standard operating procedures (SOPs), as applicable.

The manufacturer will provide the principal investigator with an up-to-date Investigator's Brochure (IB) in addition to the protocol.

The principal investigator will permit trial-related monitoring, audits, IRB and IBC review, and regulatory inspections, providing direct access to source data/documents.

Audits
Quality assurance (QA) or other designated personnel (i.e., independent auditing company) may carry out audits for which the principal investigator and staff must provide support at all times. The purpose of the audit is to ensure that ethics, regulatory and quality requirements are fulfilled.

Financial agreements and disclosure
A clinical study agreement will be signed by the principal investigator (and/or, as appropriate, the hospital administrative representative), as well as the manufacturer prior to the start of the study. The agreement will outline overall manufacturer and principal investigator responsibilities in relation to the study.

16.2 Data and Safety Monitoring

The Data and Safety Monitoring (DSM) Plans at Memorial Sloan-Kettering Cancer Center were approved by the National Cancer Institute in September 2001. The plans address the new policies set forth by the NCI in the document entitled "Policy of the National Cancer Institute for Data and Safety Monitoring of Clinical Trials" which can be found at: http://cancertrials.nci.nih. gov/researchers/dsm/index.html. The DSM Plans at MSKCC were established and are monitored by the Office of Clinical Research. The MSKCC Data and Safety Monitoring Plans can be found on the MSKCC Intranet at: *http://mskweb2.mskcc.org/irb/index.htm.*

There are several different mechanisms by which clinical trials are monitored for data, safety and quality. There are institutional processes in place for quality assurance (e.g., protocol monitoring, compliance and data verification audits, therapeutic response, and staff education on clinical research, QA) and departmental procedures for quality control, plus there are two institutional committees that are responsible for monitoring the activities of our clinical trials programs. The committees: *Data and Safety Monitoring Committee (DSMC)* for Phase I and II clinical trials, and the *Data and Safety Monitoring Board (DSMB)* for Phase III clinical trials, report to the Center's Research Council and Institutional Review Board.

During the protocol development and review process, each protocol will be assessed for its level of risk and degree of monitoring required. Every type of protocol (e.g., NIH sponsored, in-house sponsored, industrial sponsored, NCI cooperative group, etc.) will be addressed and the monitoring procedures will be established at the time of protocol activation.

The MSKCC Data and Safety Monitoring Committee (DSMC) will monitor this trial. The activities of the board will include a regular review of all relevant clinical data, regular meetings and statements on the safety of the study participants, and specifically the decision on enrolling patients into the next higher dose level.

16.3 Data Handling and Statistical Methods

Data collection and data handling procedures

The principal investigator and/or research team is required to prepare and maintain adequate and accurate case histories designed to record all observations and other data pertinent to the study for each study participant. All information recorded in CRDB for this study must be consistent with the patient's source documentation (i.e. medical records).

Drop-outs and missing data
All patient withdrawals and their reasons for withdrawal will be recorded. All data from patients who withdraw (e.g., who are discontinued prematurely) will be documented and discussed, as necessary, in the final report of the trial. Missing data will be dealt with on an individual basis. The data of eligible patients who withdraw after the screening examination, but prior to treatment, may be listed but not analyzed otherwise.

In general, all available data will be included in the analyses and will be summarized as far as possible. All missing data will be subject to data queries as specified above. There will be no substitution of missing data, i.e., missing data will not be replaced and will be handled as 'missing' in the statistical evaluation. In the case of a premature termination, the last available observation will be used for analysis (LOCF approach), if applicable. Missing baseline data will not be replaced. As agreed upon in writing with the supplier of GL-ONC1, clinical trial data will also be provided to Genelux Corporation in support of regulatory filings providing additional data for exposure of patients to GL-ONC1.

Source documentation
The principal investigator, and/or research team, is required to prepare and maintain adequate and accurate documentation for the study and to record all observations and other data pertinent to the study for each study participant. Source documents are original documents, data and records (e.g., hospital records, clinical and office charts, laboratory notes, memoranda, patients' diaries or evaluation checklists, pharmacy dispensing records, recorded data from automated instruments, copies or transcriptions certified after verification as being accurate copies, microfiches, photographic negatives, microfilm or magnetic media, X-rays, patient files, and records kept at the pharmacy, at the laboratories and at medico-technical departments involved in the clinical trial).

Source documents should be clearly marked and permit easy identification of participation by an individual in the specified clinical trial.

Whenever a patient name is revealed on a document that may be reviewed by a designated person not

affiliated with the institution (e.g., CRA) , the name and other private health information according to the Health Insurance Portability and Accountability Act (1996) (HIPAA) must be blacked out permanently by the site personnel and annotated with the patient's trial-specific number as identification.

Storage of study records

Essential documents should be retained for at least two years after the last approval of a marketing application in an ICH-GCP region, and until there are no pending or planned marketing applications in an ICH-GCP region, or until at least two years have elapsed since the formal discontinuation of clinical development of the product by the manufacturer. These documents should be retained for a longer period, however, if required by the applicable regulatory requirements or by an agreement with the manufacturer. The principal investigator/institution should take measures to prevent accidental or premature destruction of these documents. The archiving arrangements will be addressed by the CRA when closing-out the site. The manufacturer will inform the principal investigator, in writing, as to when these documents are no longer required to be retained. No documents are to be destroyed until the principal investigator is notified by Genelux Corporation.

If the principal investigator relocates or retires, or otherwise withdraws his/her responsibility for maintenance and retention, the manufacturer must be notified in writing so that adequate provision can be made with regard to the trial documents.

16.4 Institutional Review Board (IRB) and Institutional Biosafety Committee (IBC) approvals

The study protocol is approved by the manufacturer, the principal investigator, the IRB and IBC at the principal investigator's site. The principal investigator will ensure that these requirements are met before any patients are enrolled in the study.

The principal investigator will not deviate from this protocol for any reason except in cases of medical emergencies, or when the change is necessary to eliminate an apparent immediate hazard to the patient. In that event, the principal investigator will notify the MSKCC IRB. Protocol waivers are not allowed and will not be approved, if sought. Exceptions to eligibility criteria will also not be granted.

Documented cycle delays that occur outside of the allowable window of variance (± 3 days) due to holidays, weekends, weather, or other unforeseen circumstances will not constitute a protocol deviation.

After the protocol has been submitted to the IRB and IBC, any change will require a formal amendment.

The IRB and the IBC must approve of all amendments. Approval must also be obtained from the regulatory authorities, as required.

The principal investigator will provide notification to the manufacturer of amendment approvals by regulatory authorities, if applicable. The principal investigator is required to supply the manufacturer with all IRB and IBC documentation received on behalf of a protocol amendment submission.

17.0 PROTECTION OF HUMAN SUBJECTS

Good Clinical Practice and Declaration of Helsinki

The procedures set out in this study protocol are designed to ensure that the principal investigator abides by the principles of the ICH Guideline for Good Clinical Practice (GCP E6) and the Declaration of Helsinki concerning the conduct, evaluation and documentation of the study. The study will also be performed adhering to the state, local and federal legal conditions and regulations and to the applicable regulatory requirements. The principal investigator must confirm this by signing the study protocol.

Inclusion of Children in Research

This protocol/project does not include children because the number of children is limited and because the majority are already accessed by a nationwide pediatric cancer research network. This statement is based on exclusion 4b of the NIH Policy and Guidelines on the Inclusion of Children as Participants in Research Involving Human Subjects.

17.1 Privacy

MSKCC's Privacy Office may allow the use and disclosure of protected health information pursuant to a completed and signed Research Authorization form. The use and disclosure of protected health information will be limited to the individuals described in the Research Authorization form. A Research Authorization form must be completed by the Principal Investigator and approved by the IRB and Privacy Board (IRB/PB).

17.2 Serious Adverse Event (SAE) Reporting

An adverse event is considered serious if it results in ANY of the following outcomes:

- Death
- A life-threatening adverse event
- An adverse event that results in inpatient hospitalization or prolongation of existing hospitalization
- A persistent or significant incapacity or substantial disruption of the ability to conduct normal life functions
- A congenital anomaly/birth defect
- Important Medical Events (IME) that may not result in death, be life threatening, or require hospitalization may be considered serious when, based upon medical judgment, they may jeopardize the patient or subject and may require medical or surgical intervention to prevent one of the outcomes listed in this definition

Note: Hospital admission for a planned procedure/disease treatment is not considered an SAE.

SAE reporting is required as soon as the participant signs consent. SAE reporting is required for 30-days after the participant's last investigational treatment or intervention. Any events that occur after the 30-day period and that are at least possibly related to protocol treatment must be reported.

If an SAE requires submission to the IRB office per IRB SOP RR-408 'Reporting of Serious Adverse Events', the SAE report must be sent to the IRB within 5 calendar days of the event. The IRB requires a Clinical Research Database (CRDB) SAE report be submitted electronically to the SAE Office as follows:

For IND/IDE trials: Reports that include a Grade 5 SAE should be sent to saegrade5@mskcc.org. All other reports should be sent to saemskind@mskcc.org.

For all other trials: Reports that include a Grade 5 SAE should be sent to saegrade5@mskcc.org. All other reports should be sent to sae@mskcc.org.

The report should contain the following information:

Fields populated from CRDB:

- Subject's initials
- Medical record number

- Disease/histology (if applicable)
- Protocol number and title

Data needing to be entered:

- The date the adverse event occurred
- The adverse event
- The grade of the event
- Relationship of the adverse event to the treatment (drug, device, or intervention)
- If the AE was expected
- The severity of the AE
- The intervention
- Detailed text that includes the following
    - A explanation of how the AE was handled
    - A description of the subject's condition
    - Indication if the subject remains on the study
- If an amendment will need to be made to the protocol and/or consent form
- If the SAE is an Unanticipated Problem

The PI's signature and the date it was signed are required on the completed report.

For IND/IDE protocols:
The CRDB SAE report should be completed as per above instructions. If appropriate, the report will be forwarded to the FDA by the SAE staff through the IND Office.

17.2.1

17.2.1

All deaths on study must be reported to the IRB and SAE Manager using expedited reporting regardless of causality. Attribution to treatment or other cause should be provided.

17.3 Adverse Events

An adverse event (AE) is defined as any untoward medical occurrence in a clinical investigation subject administered a pharmaceutical product, at any dose, that does not necessarily have to have a causal relationship with this treatment. An AE can therefore be any unfavorable and unintended sign (including abnormal laboratory findings), symptom, or disease temporally associated with the use of a medicinal product, whether or not considered related to the medicinal product. This definition includes intercurrent illnesses or injuries, and exacerbation of pre-existing conditions.

Where a diagnosis is possible, it is preferable to record it on the CRF rather than a series of terms relating to the diagnosis.

All AEs will be monitored until resolution or, if the AE is determined to be chronic, a cause is identified. If an AE remains unresolved at the conclusion of the study, the principal investigator and medical monitor will make a clinical assessment whether continued follow-up of the AE is warranted.

17.4 Serious Adverse Events

An SAE is defined as any untoward medical occurrence that, at any dose:
- Results in death.

- Is life threatening, i.e., in the opinion of the principal investigator, the AE places the patient at immediate risk of death from the event as it occurred; it does not include a reaction that, had it occurred in a more severe form, might have caused death.
- Requires inpatient hospitalization or prolongation of an existing hospitalization.
- Results in persistent or significant disability or incapacity.
- Is a congenital anomaly or birth defect.
- Is an important and significant medical event that, based upon appropriate medical judgment, may jeopardize the patient or may require medical or surgical intervention to prevent one of the other outcomes listed above.

17.5 Unexpected Adverse Event

An unexpected AE is one of a type not consistent in nature or severity with information in the current Investigator's Brochure.

17.6 Anticipated adverse events for GL-ONC1

Based on currently known safety data from another phase I clinical trial using GL-ONC1, Genelux does not anticipate that any serious adverse events will occur related to administration of GL-ONC1. However, the following conditions, similar to cold or flu symptoms, are known to be associated with vaccinia viruses in general. These events will be monitored specifically.

Systemic Effects	Local Injection
• fever<br>• rigors/chills<br>• nausea<br>• fatigue/lethargy<br>• sweating<br>• rash/ulceration<br>• myalgia<br>• lymph node swelling<br>• asthenia<br>• headache<br>• vomiting<br>• high or low blood pressure<br>• Elevated liver function tests (AST, ALT & alkaline phosphatase)	• redness<br>• injection site reaction<br>• swelling<br>• pustule formation<br>• pain<br>• rash/ulceration

17.7 Benefit-risk assessment

There is a great value and need to investigate new treatment options such as GL-ONC1. Due to its special features, GL-ONC1 could have the potential benefit of direct oncolytic destruction of tumor cells, as well as by an indirect attack of the primary tumor by a possible generation of a long-lasting anti-tumor immunity.

Potential risks related to the treatment with GL-ONC1 include flu-like symptoms after administration, pain, tissue and skin irritation at the site of peritoneal catheter insertion, systemic inflammatory immune reaction, nausea and vomiting, headache, hypotension, and an allergic reaction.

Unexpected serious harmful side effects are possible. In this case, rescue medication is available on the ward, intensive care units are reachable within minutes, and in case of a systemic vaccinia infection, anti-vaccinia antidote therapy is available.

17.8 Determination of Severity

The principal investigator will determine the severity of events reported using CRDB as follows:

- Mild: No limitation of usual activities
- Moderate: Some limitation of usual activities
- Severe: Inability to carry out usual activities
- Life threatening: Immediate hazard to life

17.9 Determination of Causality

The principal investigator will determine the relationship of the study treatment to an AE based on the following definitions:

Not Related:

- The AE is not related if exposure to the IP has not occurred or,
- The occurrence of the AE is not reasonably related in time or,
- The AE is considered unlikely to be related to use of the IP (i.e., there are no facts [evidence] or arguments to suggest a causal relationship).

Unlikely Related:

- An AE whose time relationship to IP administration makes a causal connection improbable, but which could be plausibly explained by underlying disease or other drugs or chemicals.

Unassessable:

- An AE with insufficient information to permit assessment and identification of the cause.

Possibly Related:

- The IP administration and the AE are reasonably related in time and,
- The AE could be explained equally well by factors or causes other than exposure to the IP.

Probably Related:

- The IP administration and the AE are reasonably related in time and,
- The AE could be explained equally well by factors or causes other than exposure to the IP.

Definitely Related:

- The IP administration and the AE are related in time and,
- The AE is explained by exposure to the IP and,
- No other factors or causes cannot be considered as explanations for the AE.

AEs and SAEs must be reported by using the CRDB. Follow-up of adverse events must also be done in the CRDB,

17.10 Recording and Reporting Adverse Events

Prior to enrolment of any patient in this study, the procedures for reporting serious adverse events will be reviewed with the principal investigator and research team. These procedures will include, but are not restricted to, informing the IRB at MSKCC
All adverse events are recorded in CRDB.

In addition to the institution AE reporting requirements, the following categories of adverse events must be reported to SAE Manager within 24 hours (one working day) of discovery by the principal investigator and the research team:

1. Serious Adverse Events (SAEs).
2. All adverse events requiring the withdrawal of a patient from the study.
3. All pregnancies identified during the reporting period.

Adverse events requiring reporting within 24 hours must be sent by to the SAE Manager

At the time the adverse event is initially reported to the SAE Manager, the principal investigator will be asked to supply detailed information regarding the nature and severity of the event including, but not limited to:

- Patient ID number;
- Date and time of GL-ONC-1 treatment;
- Start and stop date (if known);
- Maximum intensity of the event (CTCAE Vers. 4 grading);
- Likelihood of its relationship to the study medication;
- Treatment administered as a result of the event;
- Any concomitant medications taken before or as a result of the event;

SAE information will be added to the Investigator's Brochure on a minimum annual basis, as appropriate.

Follow-up of Adverse Events

The principal investigator is responsible for ensuring that all AEs identified during the reporting period are followed until they are resolved or until they become chronic with no resolution expected. Follow-up of all AEs must be documented in the patient's medical record and CRDB.

18.0 INFORMED CONSENT PROCEDURES

Before protocol-specified procedures are carried out, consenting professionals will explain full details of the protocol and study procedures as well as the risks involved to participants prior to their inclusion in the study. Participants will also be informed that they are free to withdraw from the study at any time. All participants must sign an IRB/PB-approved consent form indicating their consent to participate. This consent form meets the requirements of the Code of Federal Regulations and the Institutional Review Board/Privacy Board of this Center. The consent form will include the following:

1. The nature and objectives, potential risks and benefits of the intended study.
2. The length of study and the likely follow-up required.
3. Alternatives to the proposed study. (This will include available standard and investigational therapies. In addition, patients will be offered an option of supportive care for therapeutic studies.)
4. The name of the investigator(s) responsible for the protocol.
5. The right of the participant to accept or refuse study interventions/interactions and to withdraw from participation at any time.

Before any protocol-specific procedures can be carried out, the consenting professional will fully explain the aspects of patient privacy concerning research specific information. In addition to signing the IRB Informed Consent, all patients must agree to the Research Authorization component of the informed consent form.

Each participant and consenting professional will sign the consent form. The participant must receive a copy of the signed informed consent form.

19.0 REFERENCES

1. Kawa A, Arakawa S. The effect of attenuated vaccinia virus AS strain on multiple myeloma; a case report. *Jpn J Exp Med.* 1987;57(1):79-80.
2. Arakawa S Jr, Hamami G, Umezo K, Kamidono S, Ishigami J, Arakawa S. Clinical trial of attenuated vaccinia virus AS strain in the treatment of advanced adenocarcinoma. Report on two cases. *J Cancer Res Clin Oncol.* 1987;113(1):95-8.
3. Hansen RM, Libnoch JA. Remission of chronic lymphatic leukemia after smallpox vaccination. *Arch Intern Med.* 1978;138(7):1137-8.
4. Yettra M. Remission of chronic lymphocytic leukemia after smallpox vaccination. *Arch Intern Med.* 1979;139(5):603.
5. Lane JM, Ruben FL, Neff JM, Millar JD. Complications of smallpox vaccination, 1968: results of ten statewide surveys. *J Infect Dis* 1970;122:303-9.
6. Lane JM, Ruben FL, Neff JM, Millar JD. Complications of smallpox vaccinations, 1968: national surveillance in the United States. *New Engl J Med* 1969;281:1201-8.
7. Mastrangelo MJ, Lattime EC. Virotherapy clinical trials for regional disease: *in situ* immune modulation using recombinant poxvirus vectors. *Cancer Gene Ther.* 2002;9(12):1013-21.
8. Gomella LG, Mastrangelo MJ, McCue PA, Maguire HC JR, Mulholland SG, Lattime EC. Phase I study of intravesical vaccinia virus as a vector for gene therapy of bladder cancer. *J Urol*. 2001;166(4):1291-5.
9. Mastrangelo MJ, Maguire HC, Lattime EC. Intralesional vaccinia/GM-CSF recombinant virus in the treatment of metastatic melanoma.
10. Mastrangelo MJ, Maguire HC Jr, Eisenlohr LC, Laughlin CE, Monken CE, McCue PA, Kovatich AJ, Lattime EC. Intratumoral recombinant GM-CSF-encoding virus as gene therapy in patients with cutaneous melanoma. *Cancer Gene Ther*. 1999;6(5):409-22.
11. Liu TC, Hwang T, Park BH, Bell J, Kirn DH. The targeted oncolytic poxvirus JX-594 demonstrates antitumoral, antivascular, and anti-HBV activities in patients with hepatocellular carcinoma. *Mol Ther. 2008*;16(9):1637-42.
12. Park BH, Hwang T, Liu TC, Sze DY, Kim JS, Kwon HC, Oh SY, Han SY, Yoon JH, Hong SH, Moon A, Speth K, Park C, Ahn YJ, Daneshmand M, Rhee BG, Pinedo HM, Bell JC, Kirn DH. Use of a targeted oncolytic poxvirus, JX-594, in patients with refractory primary or metastatic liver cancer: a phase I trial. *Lancet Oncol*. 2008;9(6):533-42.
13. Kelly KJ, Woo Y, Brader P, Yu Z, Riedl C, Lin SF, Chen N, Yu YA, Rusch VW, Szalay AA, Fong Y. Novel oncolytic agent GLV-1h68 is effective against malignant pleural mesothelioma. *Hum Gene Ther*. 2008;19(8):774-82.
14. Buller et al., (1985), In: Quinnan, G.V. ed. Vaccinia Viruses as Vectors for Vaccine Antigens, New York: Elsevier, 37-46
15. Byrne MJ, Nowak AK. Modified RECIST criteria for assessment of response in malignant pleural mesothelioma. *Ann Oncol.* 2004;15(2):257-60.
16. Donat U, Weibel S, Hess M, Stritzker J, Hartl B, et al. (2012) Preferential Colonization of Metastases by

Oncolytic Vaccinia Virus Strain GLV-1h68 in a Human PC-3 Prostate Cancer Model in Nude Mice. *PLoS ONE*.7(9): e45942. Doi:10.1371/journal.pone.0045942

17. Burshtyn, DN (2013) NK cells and poxvirus infection. Frontiers in Immunology. 2013 7(4): doi:10.3389/fimmu.2013.00007

18. Breitbach CJ, DeSilva NS, Falls TJ, Aladl U, Evgin L, Paterson J, Sun YY, Roy DG, Rintoul, JL, Daneshmand M, Parato K, Stanford MM, Lichty BD, Fenster A, Kirn D, Atkins H, & Bell JC. Targeting Tumor Vasculature With Oncolytic Virus. *The American Society of Gene & Cell Therapy*. 5(19): 886-894.

19. Lin et al. Vaccinia for Anaplastic Thyroid Cancer. *J Clin Endocrinol Metab*, November 2008, 93 (11):4403-4407

20.0 APPENDICES

Appendices

Appendix 1 Study Calendar
Appendix 2 Biological Sample Processing and Shipment Manual
Appendix 3 Treatment Guidelines for Use of Vaccinia Immune Globulin (VIG)
Appendix 4 Summary of Vaccinia-related Adverse Events
Appendix 5 Preparation of Patient Samples and Analysis by Vaccinia Virus Plaque Assay
Appendix 7 Assay for determination of vaccinia virus specific antibodies in serum/plasma samples from patients by determination of 50% plaque reduction titer
Appendix 8 Drug Handling Guidelines
Appendix 9 GL-ONC1 Chain of Custody
Appendix 10 Accidental Vaccina Virus Exposure
Appendix 11 Protocol for Immunohistochemical staining of GL-ONC1
Appendix 12 Quantification of Beta-glucuronidase

Study Tools

1. **VNS and Caretaker Information Letter**
